# Metformin has anti-inflammatory effects and induces immunometabolic reprogramming via multiple mechanisms in hidradenitis suppurativa

**DOI:** 10.1093/bjd/ljad305

**Published:** 2023-08-30

**Authors:** Andreea Petrasca, Roisin Hambly, Niamh Kearney, Conor M Smith, Emily K Pender, Julie Mac Mahon, Aoife M O’Rourke, Mohamed Ismaiel, Patrick A Boland, Jose P Almeida, Czara Kennedy, Alexandra Zaborowski, Siun Murphy, Desmond Winter, Brian Kirby, Jean M Fletcher

**Affiliations:** School of Biochemistry and Immunology, Trinity Biomedical Sciences Institute, Trinity College Dublin, Dublin, Ireland; Department of Dermatology, St. Vincent’s University Hospital, Dublin, Ireland; Charles Institute of Dermatology, Dublin, Ireland; Department of Dermatology, St. Vincent’s University Hospital, Dublin, Ireland; Charles Institute of Dermatology, Dublin, Ireland; School of Biochemistry and Immunology, Trinity Biomedical Sciences Institute, Trinity College Dublin, Dublin, Ireland; Department of Dermatology, St. Vincent’s University Hospital, Dublin, Ireland; Charles Institute of Dermatology, Dublin, Ireland; Department of Dermatology, St. Vincent’s University Hospital, Dublin, Ireland; Charles Institute of Dermatology, Dublin, Ireland; School of Biochemistry and Immunology, Trinity Biomedical Sciences Institute, Trinity College Dublin, Dublin, Ireland; Department of Surgery, St. Michael’s Hospital, Dublin, Ireland; Department of Surgery, St. Michael’s Hospital, Dublin, Ireland; Department of Surgery, St. Michael’s Hospital, Dublin, Ireland; Department of Surgery, St. Michael’s Hospital, Dublin, Ireland; Department of Surgery, St. Michael’s Hospital, Dublin, Ireland; Department of Plastic Reconstructive and Aesthetic Surgery, Blackrock Clinic, Dublin, Ireland; Department of Surgery, St. Michael’s Hospital, Dublin, Ireland; Department of Dermatology, St. Vincent’s University Hospital, Dublin, Ireland; Charles Institute of Dermatology, Dublin, Ireland; School of Biochemistry and Immunology, Trinity Biomedical Sciences Institute, Trinity College Dublin, Dublin, Ireland; School of Medicine, Trinity Biomedical Sciences Institute, Trinity College Dublin, Dublin, Ireland

## Abstract

**Background:**

Targeting immunometabolism has shown promise in treating autoimmune and inflammatory conditions. Hidradenitis suppurativa (HS) is a chronic inflammatory skin disease involving painful lesions in apocrine gland-bearing skin. Therapeutic options for HS are limited and often ineffective; thus, there is a pressing need for improved treatments. To date, metabolic dysregulation has not been investigated in HS. As HS is highly inflammatory, we hypothesized that energy metabolism is dysregulated in these patients. Metformin, an antidiabetic drug, which is known to impact on cellular metabolic and signalling pathways, has been shown to have anti-inflammatory effects in cancer and arthritis. While metformin is not licensed for use in HS, patients with HS taking metformin show improved clinical symptoms.

**Objective:**

To assess the effect and mechanism of action of metformin in HS.

**Methods:**

To assess the effect of metformin *in vivo*, we compared the immune and metabolic profiles of peripheral blood mononuclear cells (PBMCs) of patients with HS taking metformin vs. those not taking metformin. To examine the effect of metformin treatment *ex vivo*, we employed a skin explant model on skin biopsies from patients with HS not taking metformin, which we cultured with metformin overnight. We used enzyme-linked immunosorbent assays, multiplex cytokine assays and quantitative real-time polymerase chain reaction (RT-PCR) to measure inflammatory markers, and Seahorse flux technology and quantitative RT-PCR to assess glucose metabolism.

**Results:**

We showed that metabolic pathways are dysregulated in the PBMCs of patients with HS vs. healthy individuals. In metformin-treated patients, these metabolic pathways were restored and their PBMCs had reduced inflammatory markers following long-term metformin treatment. In the skin explant model, we found that overnight culture with metformin reduced inflammatory cytokines and chemokines and glycolytic genes in lesions and tracts of patients with HS. Using *in vitro* assays, we found that metformin may induce these changes via the NLR family pyrin domain containing 3 (NLRP3) inflammasome and the AMP-activated protein kinase (AMPK)–mammalian target of rapamycin (mTOR) pathway, which is linked to glycolysis and protein synthesis.

**Conclusions:**

Our study provides insight into the mechanisms of action of metformin in HS. The anti-inflammatory effects of metformin support its use as a therapeutic agent in HS, while its effects on immunometabolism suggest that targeting metabolism is a promising therapeutic option in inflammatory diseases, including HS.

## Teaser

Our study provides insight into the mechanisms of action of metformin in hidradenitis suppurativa (HS). The anti-inflammatory effects of metformin support its use as a therapeutic agent in HS, while its effects on immunometabolism suggest that targeting metabolism is a promising therapeutic option in inflammatory diseases, including HS.

## Graphical Abstract

**Graphical Abstract:**
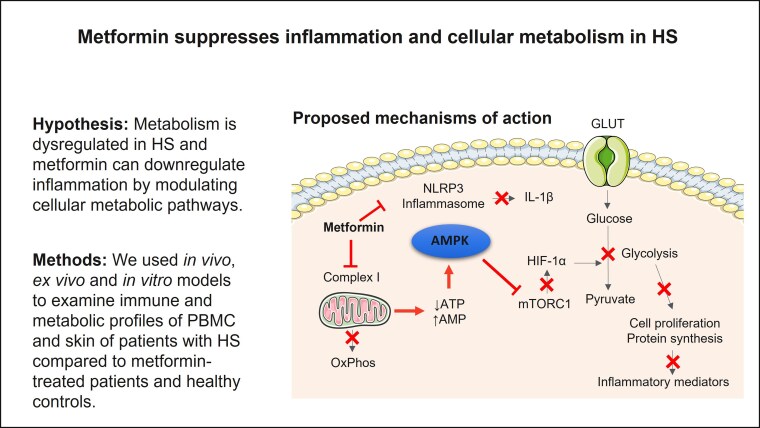

Linked Article: Melnik *Br J Dermatol* 2023; **189**:652–653.

Plain language summary available online

Author Video: https://youtu.be/AhbdvDhbVek?feature=shared

**What is already known about this topic?**

- The pathogenesis of hidradenitis suppurativa (HS) is not fully understood, but it involves an interplay between the skin and immune cells, resulting in an inflammatory loop, fuelling tissue destruction.
- Biologic treatments are limited and not fully effective, and there is a pressing unmet need for improved treatment options.
- Metformin, an antidiabetic drug that is used off-label to treat HS, was shown to improve clinical symptoms in several clinical series.

**What does this study add?**

- This study demonstrates that metabolism is dysregulated in HS, which may contribute to inflammation.
- This study shows that metformin suppresses inflammation and regulates metabolic pathways in patients with HS.
- The results indicate that targeting immunometabolism in HS has anti-inflammatory effects and is thus a promising therapeutic option.
- The findings offer insight into the mechanisms of action of metformin in HS.

**What is the translational message?**

- Drugs targeting immunometabolism are currently in trials for their anti-inflammatory properties and could be tested in HS.
- The anti-inflammatory effects of metformin provide rationale for its use in treating HS.

Hidradenitis suppurativa (HS) is a debilitating chronic inflammatory skin disease characterized by recurrent lesions in apocrine gland-bearing skin.^1^ The pain, discomfort and location of the lesions result in poor quality of life, which is further aggravated by comorbidities such as diabetes and inflammatory bowel disease.^2^ The pathogenesis of HS is not fully understood, but it involves abnormal immune function and follicular obstruction. It is unclear which is the primary event, but the result is immune activation and infiltration of immune cells. Secretion of inflammatory mediators further perpetuates inflammation by activating keratinocytes and fibroblasts, which recruit immune cells, maintaining an inflammatory loop that contributes to tissue destruction and scarring.^3,4^ Current treatment options for HS, which include surgical intervention, are limited and not fully effective, often resulting in recurrence.^5^ Adalimumab, the only US Food and Drug Administration-approved biologic for HS, has been found to reduce inflammatory lesions by at least 50% in about 50% of patients.^6^ Hence, there is a pressing need for a better understanding of HS pathogenesis to underpin the development of novel and more efficacious therapeutics.

Dysregulated cellular metabolism is a key feature of inflammatory diseases, including arthritis and some cancers.^7,8^ Akin to the Warburg effect described in cancer cells, immune cells can undergo metabolic reprogramming, switching to glycolysis during immune activation to produce energy to sustain the increased metabolic demand required for cellular proliferation and effector functions.^9^ Thus, targeting metabolic processes through immunometabolic reprogramming is a promising new therapeutic option for inflammatory diseases,^10^ with longstanding drugs, including dimethyl fumarate, metformin, methotrexate and rapamycin, now thought to exert their anti-inflammatory properties in inflammatory diseases – at least in part – by targeting immunometabolism.^10,11^ To date, cellular energetic pathways have yet to be investigated in HS.

Metformin, the first-line drug for type 2 diabetes mellitus (T2DM), has been demonstrated to have anti-inflammatory effects in cancers and inflammatory diseases by altering cellular metabolic and signalling pathways,^12,13^ and has been reported in a number of case series to be effective in HS.^14,15^ While the mechanism of action of metformin in HS is unknown, we hypothesized that it reduces inflammation, at least in part, by modulating metabolic pathways. In this study, we investigated the effects of metformin in patients with HS *in vivo* and *ex vivo*. We found that long-term metformin treatment *in vivo* resulted in reduced inflammatory mediators and glycolysis in the blood of patients with HS. Similarly in the skin, overnight *ex vivo* treatment with metformin exerted anti-inflammatory effects and induced metabolic reprogramming. Finally, we demonstrated that metformin can modulate the AMP-activated protein kinase (AMPK)-mechanistic target of rapamycin (mTOR) pathway and inhibit the NLR family pyrin domain containing 3 (NLRP3) inflammasome in patients with HS, providing insight into its mechanism of action.

## Materials and methods

### Patient recruitment

HS blood and psoriasis skin biopsies were obtained from patients recruited at dermatology clinics at St. Vincent’s University Hospital, Dublin, Ireland. HS skin biopsies (normal-appearing uninvolved skin > 10 cm from the active lesion; lesional skin from the leading edge of the active nodules; and tracts from inflamed sinus tracts) were acquired during surgical consultation at St. Michael’s Hospital, Dublin, Ireland. Healthy donor blood was obtained from St. Vincent’s University Hospital and the Irish Blood Transfusion Service, while healthy skin was donated by volunteers undergoing mammoplasty or abdominoplasty at Blackrock Clinic, Dublin, Ireland. Participant demographics are provided in detail in Tables S1–S4 (see Supporting Information). As HS occurs in women twice as often as in men, our patients were predominantly female. Owing to the small patient numbers in this study, we could not stratify the results by sex, which is a limitation of the study. Full details of the laboratory methods employed to carry out this study, including sequences of the primers used (Table S5) are available in Appendix S1 (see Supporting Information).

### Statistical analysis

Statistical analyses were performed using Prism 9 (GraphPad, San Diego, CA, USA). Normality tests were used to identify parametric data. Mann–Whitney tests were used for unpaired comparisons, Wilcoxon tests for paired comparisons and two-way Anova with multiple comparisons tests for multiple comparisons. A *P*-value < 0.05 was considered to be statistically significant.

## Results

### Metformin reduces inflammatory cytokines

**Figure 1:**
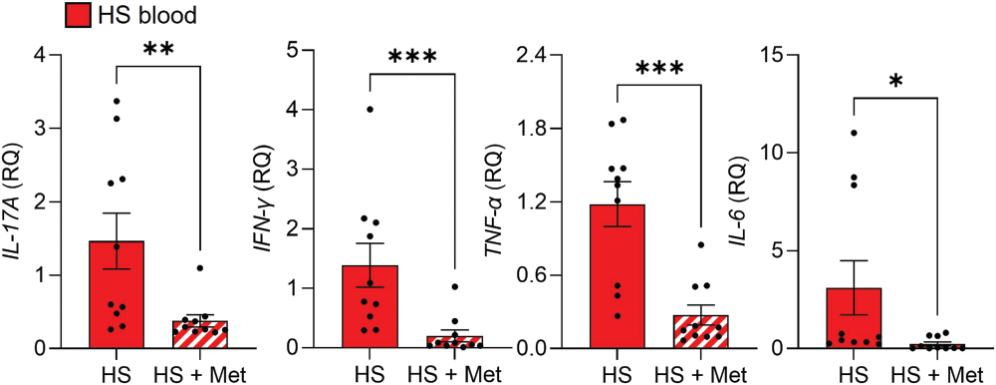
Reduced expression of inflammatory cytokines in peripheral blood mononuclear cells (PBMCs) from patients with hidradenitis suppurativa (HS) taking metformin (Met). PBMCs were isolated from metformin-naive patients with HS (*n* = 10) and patients with HS taking metformin (*n* = 10) orally for ≥ 6 months. The cells were lysed to isolate RNA, which was reverse transcribed to cDNA and analysed by quantitative real-time polymerase chain reaction for the expression of *IL17A*, *IFNG*, *TNF* and *IL6* relative to endogenous control gene *RPLP0*. Data are expressed as mean (SEM) relative quantification (RQ). *P < 0.05, **P < 0.01 and ***P < 0.001 (Mann–Whitney test).

**Figure 2:**
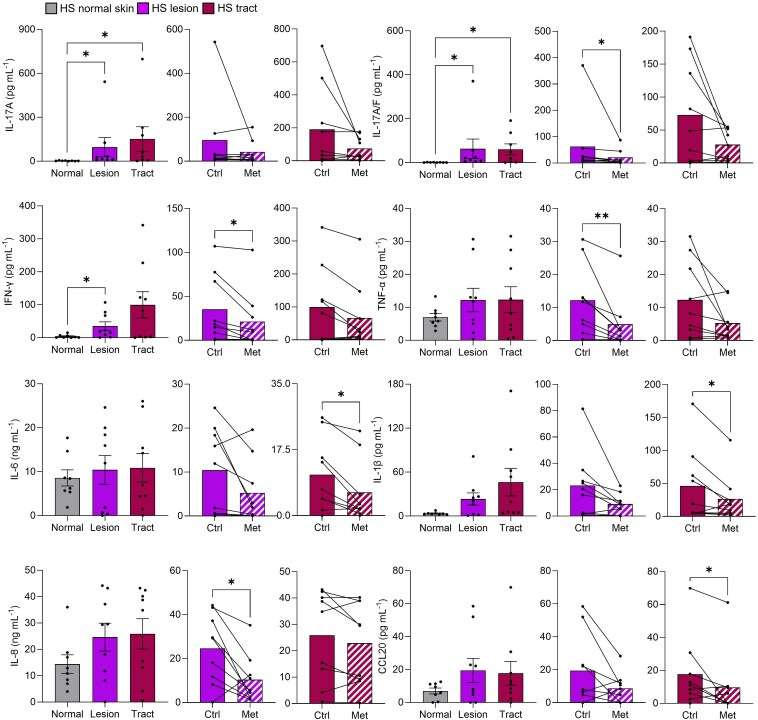
Metformin (Met) treatment *ex vivo* reduces inflammatory cytokines and chemokines in hidradenitis suppurativa (HS) skin explants. Explant cultures were set up using normal, lesion and tract samples from each patient with HS (*n* = 9) and cultured alone (Ctrl) or with metformin (5 mmol L^–1^) for 24 h. Explant conditioned media were assayed for the secretion of interleukin (IL)-17A, IL-17A/F, interferon (IFN)-γ, tumour necrosis factor (TNF)-α, IL-6, IL-1β, IL-8 and CCL20 by multiplex cytokine assay. Data are expressed as mean (SEM) concentration. *P < 0.05 and **P < 0.01 (Dunn’s multiple comparison tests or Wilcoxon matched-pair signed rank tests).

As metformin is known to have anti-inflammatory properties, we first examined its *in vivo* effects on inflammatory cytokines in the peripheral blood of patients with HS. When we compared patients taking metformin for at least 6 months with patients not on metformin and measured gene expression of inflammatory cytokines in their peripheral blood mononuclear cells (PBMCs), we found a significant reduction in interleukin (IL)-17A, interferon (IFN)-γ, tumour necrosis factor (TNF)-α and IL-6 in the metformin-treated patients (all *P* < 0.05; Figure 1). To assess the effect of metformin *ex vivo*, we used a skin explant model using lesions and tracts of patients with HS and measured the levels of inflammatory mediators after 24 h. Culture with metformin decreased the secretion of IL-17A/F, IFN-γ, TNF-α and IL-8 in lesions, and significantly reduced IL-6, IL-1β and CCL20 in tracts (all *P* < 0.05; Figure 2). When we compared normal skin biopsies with lesions and tracts of the same patients in this model, we found an increase in IL-17A, IL-17A/F and IFN-γ (all *P* < 0.05; Figure 2) vs. normal skin, as would be expected in inflammatory lesions. Psoriasis is another inflammatory skin disease that shares pathogenic features with HS. Therefore, we assessed the effects of metformin on psoriatic lesions using the same skin explant model. We found a significant reduction in IL-17A, IFN-γ, TNF-α, IL-6, IL-1α, IL-1β, IL-17C, IL-8 and CXCL1 in metformin-treated compared with untreated explants (Figure S1; see Supporting Information). These results demonstrate that metformin exerts anti-inflammatory effects in both HS and psoriasis.

### Metformin induces metabolic reprogramming

**Figure 3:**
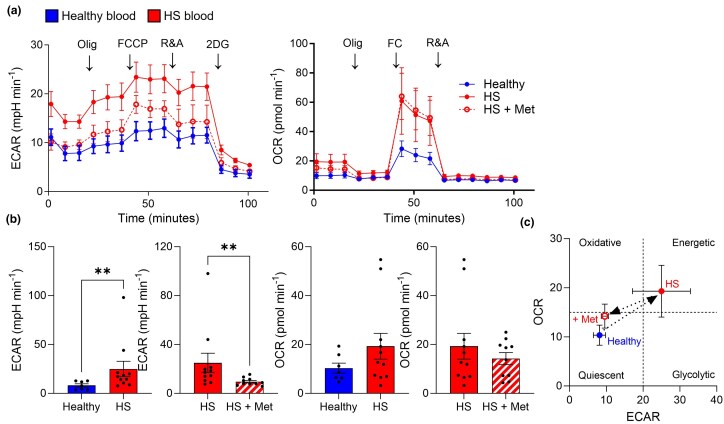
Metformin (Met)-treated patients with hidradenitis suppurativa (HS) exhibited reduced glycolysis. Peripheral blood mononuclear cells were isolated from healthy controls (*n* = 7), metformin-naive patients with HS (*n* = 11) and patients with HS taking metformin (*n* = 10) for ≥ 6 months. Cells were cultured on XFe96 plates and analysed for oxygen consumption rate (OCR, pmol min^–1^) and extracellular acidification rate (ECAR, mili pH min^–1^) by Seahorse flux analysis. (a) Representative plots of ECAR and OCR levels at baseline and after injection with oligomycin, carbonyl cyanide-p-trifluoromethoxyphenylhydrazone (FCCP), rotenone and antimycin A (R&A), and 2-deoxy-D-glucose (2-DG). (b) Mean baseline ECAR and OCR levels. (c) Phenogram depicting the metabolic shift between cohorts. Data are expressed as mean (SEM). **P < 0.01 (Mann–Whitney test).

To facilitate inflammation, cells undergo a metabolic switch to upregulate bioenergetic pathways, including glycolysis. Thus, we sought to examine whether PBMCs from patients with HS exhibited a metabolic profile distinct from healthy individuals. We measured rates of glycolysis and oxidative phosphorylation (OxPhos) using the surrogate markers extracellular acidification rate (ECAR) and oxygen consumption rate (OCR), respectively. Our results showed a significant increase in ECAR in PBMCs from patients with HS compared with those from healthy controls (*P* = 0.008), while OxPhos was unchanged (Figure 3a, b). A phenogram of OCR vs. ECAR depicted a shift from a quiescent phenotype in healthy controls to an energetic state in patients with HS (Figure 3c). When we compared patients taking metformin with those not taking it, we found a reduction in ECAR to levels similar to those of healthy controls and a return to a quiescent state (*P* = 0.007; Figure 3b, c).

**Figure 4:**
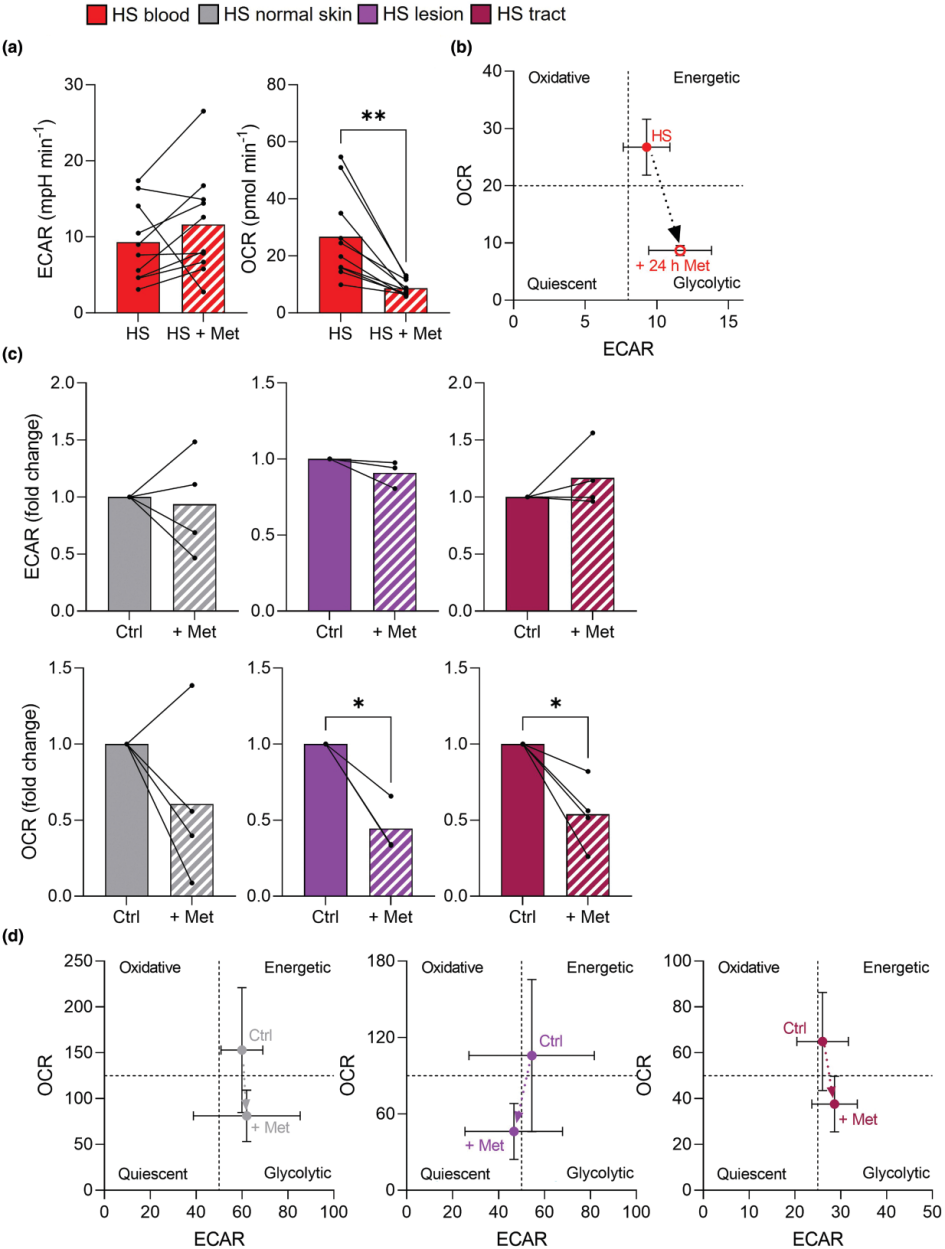
Metformin treatment *ex vivo* and *in vitro* inhibits oxidative phosphorylation in hidradenitis suppurativa (HS). To measure the effect of metformin (Met) *in vitro*, peripheral blood mononuclear cells from patients with HS not taking metformin (*n* = 10) were cultured ± metformin (5 mmol L^–1^) for 24 h. (a, b) The cells were then added to XFe96 microplates to measure baseline oxygen consumption rate (OCR) and extracellular acidification rate (ECAR) in metformin-treated vs. untreated cells. To assess the effect of metformin *ex vivo* in skin, biopsies of normal skin, lesions and tracts from patients with HS (*n* = 3/4) were placed inside XFe24 islet capture microplates. (c, d) Baseline OCR and ECAR values of skin explants were measured by Seahorse flux before (Ctrl) adding metformin (5 mmol L^–1^) for 12 h and after taking new baseline OCR and ECAR measurements. (a) Baseline ECAR and OCR levels before and after treatment with metformin for 24 h. (b) Phenogram depicting the energetic shift following *in vitro* treatment. (c) Baseline ECAR and OCR levels before and after metformin treatment *in vitro*. (d) Phenograms depicting the metabolic shift before and after metformin treatment *in vitro* in normal skin (left), lesions (centre) and tracts (right). Data are expressed as mean (SEM) fold change of baseline levels. *P < 0.05 (paired *t*-test or Wilcoxon matched-pair signed rank test).

Next, we examined the effect of metformin treatment *in vitro* using PBMCs from patients with HS not on metformin. Interestingly, 24-h culture with metformin significantly inhibited OCR and promoted a less energetic metabolic state (*P* = 0.002; Figure 4a, b). We observed a similar effect in HS explants, where metformin did not affect ECAR, but reduced OCR in lesions and tracts (*P* = 0.035 and *P* = 0.027; Figure 4c). This was also depicted by phenograms, which showed a reduction in energetic states for all biopsy types (Figure 4d). Thus, it appeared that long-term metformin use in patients fully restored cellular bioenergetics, while short-term use *in vitro* only reduced oxidative processes.

### Metformin alters expression of glycolytic genes

**Figure 5:**
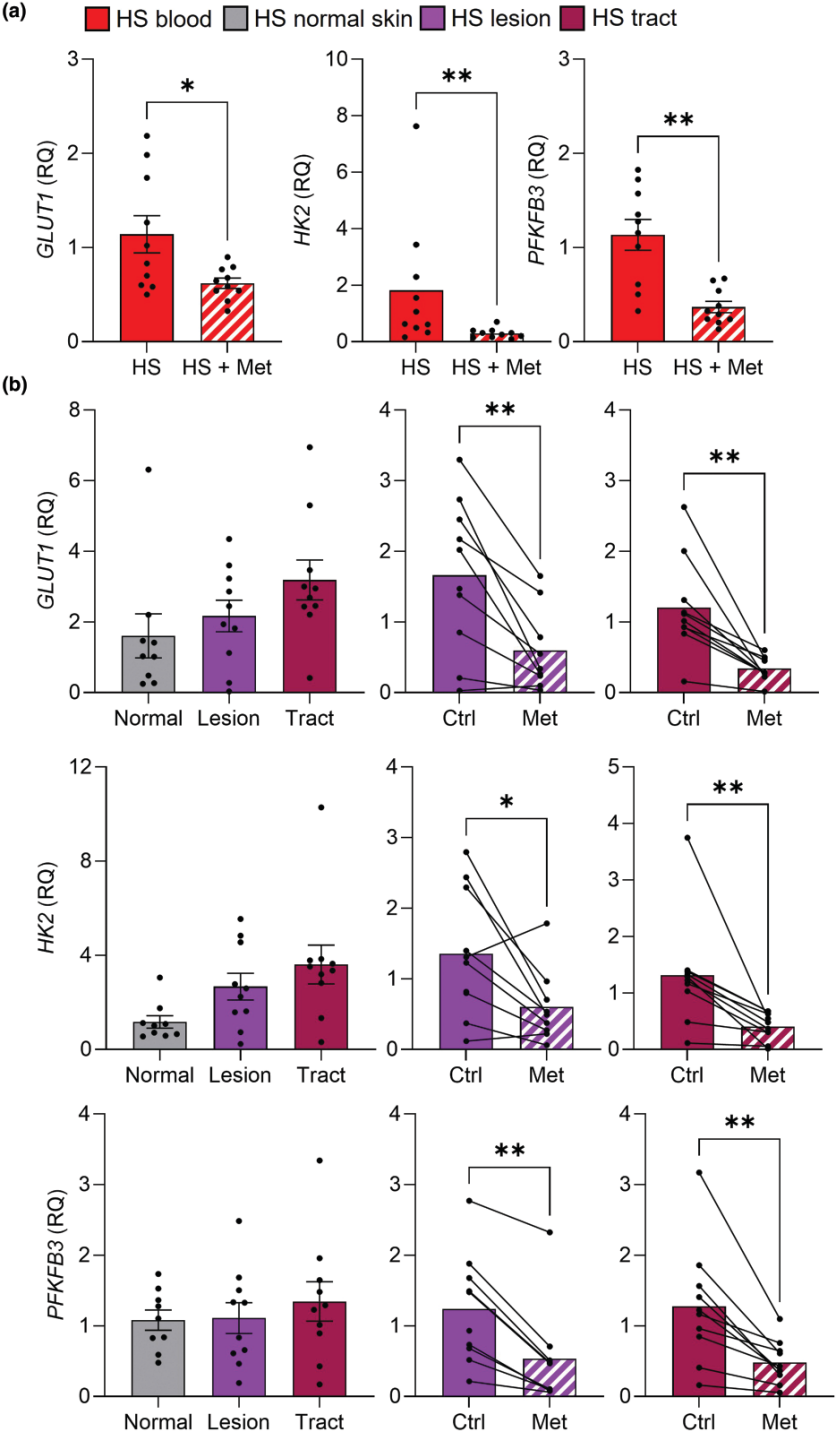
Metformin (Met) reduces glycolytic genes in the blood and skin of patients with hidradenitis suppurativa (HS). (a) Peripheral blood mononuclear cells were isolated from metformin-naive patients with HS (n = 10) and patients with HS (*n* = 10) taking metformin orally for ≥ 6 months prior to the study. (b) Biopsies of normal skin, lesions and tracts from patients with HS (*n* = 10) were cultured for 24 h alone (Ctrl) or with metformin (5 mmol L^–1^) and measured for gene expression. Cells and biopsies were lysed to isolate RNA, which was reverse transcribed to cDNA and analysed by quantitative real-time polymerase chain reaction for the expression of *GLUT1*, *HK2* and *PFKFB3* relative to endogenous control gene *RPLP0*. Data are expressed as mean (SEM) relative quantification (RQ). *P < 0.05 and **P < 0.01 (Mann–Whitney test or Wilcoxon matched-pair signed rank test).

Having measured the real-time metabolic profiles, we next measured the expression of glycolytic genes in the blood and skin of patients with HS. When examining the metformin-treated patients vs. controls, we saw a significant reduction at several points in the glycolytic pathway, including glucose transporter (*GLUT1*), hexokinase 2 (*HK2*) and phosphofructokinase B3 (*PFKFB3*) (all *P* < 0.05; Figure 5a). In HS skin explants, all three genes were also significantly decreased in lesions and tracts following treatment with metformin *ex vivo* (all *P* < 0.05; Figure 5b), demonstrating that glycolysis can be inhibited by metformin at the gene level after 24 h. When we compared patients with HS with healthy controls, *HK2* was significantly increased in HS lesions compared with healthy individuals [*P* = 0.03; Figure S2 (see Supporting Information)], indicating an increase in glycolysis.

### Mechanisms of action of metformin in hidradenitis suppurativa

**Figure 6:**
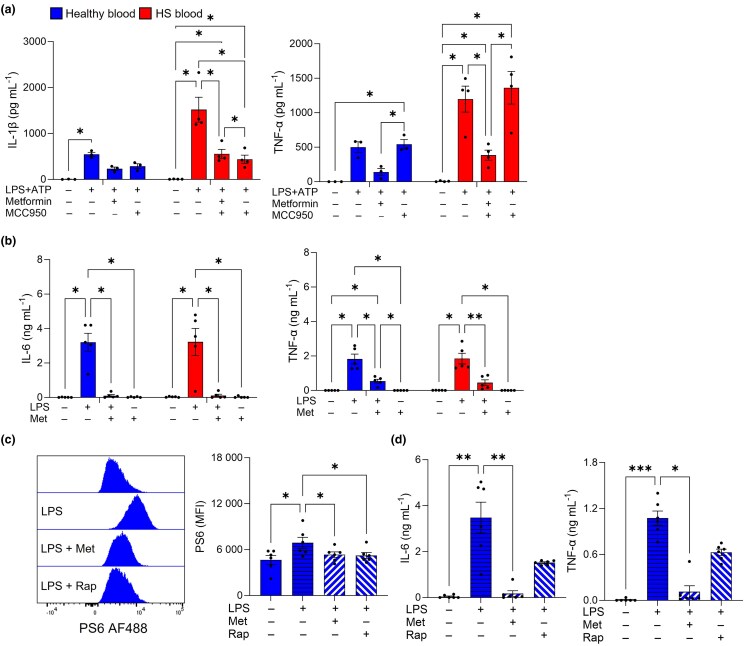
Metformin (Met) inhibits inflammasome activation and mammalian target of rapamycin (mTOR) *in vitro*. Peripheral blood mononuclear cells (PBMCs; cryopreserved) or monocytes (freshly isolated and rested overnight) were obtained from healthy controls (*n* = 5/6) and patients with hidradenitis suppurativa (HS; *n* = 5/6). PBMCs were stimulated with lipopolysaccharide (LPS; 100 ng mL^–1^) for 4 h before treating with ATP (5 mmol L^–1^) for 1 h; metformin (5 mmol L^–1^) or MCC950 (100 nmol L^–1^) was added 30 min prior to ATP. Monocytes were pretreated with metformin (5 mmol L^–1^) or rapamycin (Rap; 100 nmol L^–1^) for 30 min before stimulating with LPS (1 ng mL^–1^) for 4 h. Culture supernatants were assayed for cytokines by enzyme-linked immunosorbent assay. Monocytes were stained for extracellular expression of CD14, CD64 and viability dye, and the intracellular expression of phosphorylated ribosomal protein S6 (PS6) for flow cytometric analysis. (a) Mean concentration of interleukin (IL)-1β and tumour necrosis factor (TNF)-α from PBMCs following activation and metformin/MCC950 treatment. (b) Mean concentration of IL-6 and TNF-α from monocytes following activation and metformin/rapamycin treatment. (c) Representative histograms and bar graphs showing average median fluorescence intensity (MFI) of PS6 in monocytes. (d) Mean concentration of IL-6 and TNF-α from monocytes following metformin/rapamycin treatment. Data are expressed as mean (SEM). *P < 0.05, **P < 0.01, ***P < 0.001 and ****P < 0.0001 [two-way Anova with (a) Dunnett’s, (b) Tukey’s or (c) Dunn’s multiple comparison tests].

Having established that metformin could reduce inflammation and alter metabolic processes, we next examined its effect on innate immune activation using two models. Using an inflammasome assay where PBMCs from patients with HS or healthy controls were stimulated with lipopolysaccharide (LPS) and ATP, we found that treatment with either metformin or the known NLRP3 inflammasome inhibitor MCC950 reduced IL-1β secretion in activated PBMCs from patients with HS (*P* < 0.05; Figure 6a), while only metformin inhibited TNF-α (*P* < 0.05; Figure 6a). In LPS-stimulated monocytes, metformin treatment significantly inhibited IL-6 and TNF-α secretion in both patients with HS and healthy controls (*P* < 0.05; Figure 6b). As metformin is known to inhibit cell proliferation and protein synthesis via mTOR complex 1 (mTORC1) inhibition, we sought to investigate whether metformin-induced inhibition of monocyte cytokine production is mediated by mTORC1. mTORC1 activation was measured by flow cytometric analysis of phosphorylated ribosomal protein S6 (PS6). Both metformin and the mTOR inhibitor rapamycin inhibited PS6 in LPS-activated monocytes (*P* < 0.05; Figure 6c). Metformin significantly inhibited IL-6 and TNF-α in LPS-activated monocytes (*P* < 0.05; Figure 6d), whereas rapamycin resulted in a smaller reduction in these, suggesting that mTORC1 inhibition alone did not account for the ability of metformin to suppress these cytokines. Metformin is known to be an indirect agonist of AMPK,^16,17^ which is a metabolic regulator of cell response to energy changes.^18^ 5-Aminoimidazole-4-carboxamide 1-β-D-ribofuranotide (AICAR) is a direct AMPK agonist with anti-inflammatory properties in inflammatory diseases, including arthritis.^19,20^ Therefore, we used the skin explant model where HS skin biopsies were treated with AICAR, which significantly reduced IL-6, IL-8, TNF-α, IL-17C, IL-1α and CXCL1 in a fashion similar to metformin both at the gene and protein levels (Figure S3a, b; see Supporting Information). Additionally, AICAR significantly reduced *GLUT1* and *PFKFB3* (Figure S3c; see Supporting Information). Taken together, these results suggest that metformin may exert its anti-inflammatory and antiglycolytic actions via multiple mechanisms, including inhibition of the NLRP3 inflammasome and mTORC1, and activation of AMPK (summarized in Figure S4; see Supporting Information).

## Discussion

Recent studies have suggested that metformin has anti-inflammatory properties and protects against various diseases.^12^ This has been documented in cancers and arthritis, where metformin has been shown to suppress cell proliferation, induce apoptosis and inhibit proinflammatory cytokines.^17,21^ HS is a recurrent inflammatory disease characterized by painful skin lesions and systemic inflammation. Metformin is often used to treat HS and may be especially effective in mild-to-moderate disease. It also has the advantage of being well tolerated and inexpensive, and may also treat HS-associated comorbidities such as polycystic ovarian syndrome, T2DM and insulin resistance.^22,23^ Therefore, using a low-cost drug such as metformin would be clinically and financially beneficial. Metformin has also shown promise in treating acne vulgaris, a skin condition HS is often mistaken for and shares characteristics with.^24–26^ A retrospective study found metformin to be effective and well tolerated in patients with HS,^14^ while a prospective study found clinical improvement and improved quality of life in > 30% of patients with HS taking metformin.^15^ Furthermore, a recent report found a trend toward a reduction in inflammatory cytokines and chemokines in the serum of patients with HS taking metformin vs. those not taking metformin.^27^ While these studies examined the effects of metformin on clinical outcomes, they did not investigate the effects and mode of action of metformin at the cellular level.

In our study, we looked at the impact of metformin *in vivo* in patients with HS taking metformin for > 6 months, as well as *ex vivo* and *in vitro* in patients with HS not taking metformin, where skin biopsies or PBMCs, respectively, were treated with metformin for up to 24 h. Using these three models, we assessed the effects of metformin on the immune and metabolic profiles of these tissues. PBMCs from patients taking metformin experienced a reduction in IL-17A, IL-6, IFN-γ and TNF-α vs. controls, demonstrating that long-term metformin use has systemic anti-inflammatory effects, which could explain the clinical improvement observed in the clinical studies. We found a similar reduction in the skin explant model, where there was a reduction in the secretion of IL-1β, IL-17A/F, IFN-γ, TNF-α, IL-6, IL-8 and CCL20 after 24 h of metformin treatment. This corroborated our findings in metformin-treated patients and provides a rationale for the use of metformin to manage the chronic inflammation in patients with HS.

Immune activation is facilitated by an upregulation in energy pathways, including glycolysis, to sustain inflammatory processes. We found that PBMCs from patients with HS had increased rates of glycolysis vs. healthy controls. This is in line with a recent report of increased levels of the transcription factor hypoxia inducible factor (HIF)-1α in the lesions and serum of patients with HS,^28^ as HIF-1α is known to activate glycolytic enzymes.^29–31^ HIF-1α is also overexpressed in psoriasis.^32,33^ As metformin can alter energy metabolism, we examined the PBMCs of patients on long-term metformin treatment and found that these had reduced ECAR, as well as reduced glycolytic genes (*GLUT1*, *HK2* and *PFKFB3*). These glycolytic genes were also reduced by metformin in the skin explant model. Our findings in patients with HS agree with the findings of others where metformin was shown to inhibit glycolysis by inhibiting hexokinase 2 (HK2) activity.^34^ One of the main modes of action of metformin is by inhibiting complex I of the electron transport chain in the mitochondria, which shuts down OxPhos. We found that *ex vivo* or *in vitro* metformin treatment overnight inhibited OxPhos in HS skin and PBMCs, respectively, confirming the findings of others that metformin is a potent OxPhos inhibitor.^35^ Taken together, our results show that the short-term impact of metformin on metabolic pathways is to reduce OxPhos via complex I, while long-term treatment reduces glycolysis.

Inhibition of glycolysis by metformin is due to a downstream effect of complex I inhibition that increases the AMP : ATP ratio, causing activation of AMPK, an energy sensor and metabolic regulator in cells. Under energetic stress, AMPK restricts cell division, thereby limiting immune activity. Using AICAR, a direct agonist of AMPK, we were able to inhibit proinflammatory cytokines and chemokines in HS skin explants, similar to the inhibition we showed using metformin, supporting our finding that metformin-induced effects may be AMPK-dependent. This outcome was supported by findings in rheumatoid arthritis where both AICAR and metformin inhibited inflammatory cytokines in a similar manner.^20^

One of the effects of AMPK activation is inhibition of mTORC1. mTORC1 plays a role in glucose metabolism and mediates cell growth and protein synthesis.^36^ Therefore, we investigated whether our metformin-induced effects were mTORC1-mediated by using a model of LPS-stimulated monocytes. We found that metformin suppressed PS6, alongside rapamycin, thus indicating that in HS metformin may act – in part – via mTORC1 inhibition. When we measured IL-6 and TNF-α levels in this model, we observed a significant reduction with metformin, which was more potent than with rapamycin, suggesting that cytokine inhibition by metformin involves additional pathways. Importantly, rapamycin showed efficacy in HS as a combination rescue therapy,^37^ suggesting that targeting mTORC1 may be beneficial in HS. Furthermore, mTORC1 has been identified as a potential player in HS pathogenesis,^38,39^ and is linked to the production of various cytokines, including IL-17^40,41^ – a key player in inflammatory diseases, including HS.^42,43^ As mentioned above, in our study metformin inhibited T helper (Th) 17 cytokines, which may be mTOR-dependent, as demonstrated in other studies.^40,41,44^ The observed reduction in IL-6 and TNF-α may also be mediated by mTORC1 inhibition, which has been shown in human keratinocytes.^45^

IL-1β is a key player in inflammation and its release is mediated by the NLRP3 inflammasome.^46^ As the IL-1 pathway was found to be upregulated in HS,^47,48^ we measured IL-1β secretion from skin explants following metformin treatment and found it to be reduced in HS skin explants after 24 h. Furthermore, using an inflammasome assay, we found that metformin inhibited IL-1β to a similar extent as the inflammasome inhibitor MCC950, which suggests that metformin suppresses inflammation by blocking the NLRP3 inflammasome. This supports a recent study from our group which showed that inflammasome blockade reduced inflammation in HS.^47^ Metformin was also shown to have protective effects by targeting the NLRP3 inflammasome in other conditions.^16,49–51^ As the inhibition of NLRP3 activation can occur downstream of the AMPK–mTORC1 pathway,^52,53^ the two pathways may be linked in mediating the anti-inflammatory effects of metformin. Importantly, metformin can also inhibit mTORC1 via AMPK-independent pathways,^54–56^ which may amplify the mTORC1-mediated effects of metformin. Furthermore, metformin has shown efficacy in clinical trials as an anticancer drug through OxPhos inhibition, resulting in AMPK activation and downstream inhibition of mTORC1.^57^

mTORC1 is linked to glycolysis through its ability to drive HIF-1α activation, which promotes glycolytic gene expression,^58^ and through its ability to promote glucose uptake through GLUT1,^59^ indicating that targeting mTOR can inhibit glycolysis and subsequently cellular proliferation. Inhibiting mTORC1 can also inhibit cellular proliferation and survival via signal transducer and activator of transcription 3 (STAT3) inhibition.^58^ Metformin was shown to inhibit STAT3 via mTORC1, resulting in reduced tumour progression and suppressed Th17 signalling.^60,61^ The Th17 : regulatory T cell (Treg) axis, which is often dysregulated in inflammatory conditions, including HS,^42,62,63^ is regulated through glycolysis via HIF-1α and mTOR, which mediate Th17 cell differentiation and function.^64,65^ Therefore, inhibition of the mTORC1–HIF-1α pathway could suppress Th17-mediated inflammation and normalize the Th17 : Treg ratio. Obesity is a major risk factor for HS and the HS-associated comorbidity, T2DM. HIF-1α was found to be overexpressed in obese individuals, where it was linked to increased inflammation.^66^ Obesity and T2DM are both associated with increased mTORC1 signalling.^67^ These studies highlight the central role of mTORC1 in disease pathogenesis and suggest that metformin inhibition via mTORC1 may suppress inflammation via HIF-1α and STAT3.

In summary, metformin may mediate its anti-inflammatory and antimetabolic effects in HS via the AMPK pathway and mTORC1 through the influence of mTORC1 on mitochondrial respiration, energy metabolism, signalling pathways and the NLRP3 inflammasome.

This study has demonstrated that metformin can suppress inflammation by influencing the energy status of cells and provides a rationale for targeting cellular metabolism in HS to reduce inflammation. Metformin is generally well tolerated and inexpensive, and can induce anti-inflammatory effects by modulating immunometabolic pathways, making it a suitable candidate for HS management. Further investigation into HS pathogenesis in relation to signalling and metabolic pathways will help inform new treatments for this debilitating disease.

## Supplementary Material

ljad305_Supplementary_Data

## Data Availability

No datasets were generated or analysed in the current study. The data that support the findings of this study are available upon request from the corresponding author.
