# Supplementary material for: Metformin has anti-inflammatory effects and induces immunometabolic reprogramming via multiple mechanisms in hidradenitis suppurativa
Source: Br J Dermatol. 2023 Aug 30;189(6):730–40. doi: 10.1093/bjd/ljad305 (PMC13077222; doi:10.1093/bjd/ljad305)
Supplement: ljad305_Supplementary_Data [file ljad305_supplementary_data.zip › Appendix S1.docx]

**Appendix S1** Supplementary methods

**Cell isolation and stimulation**

Human PBMC were isolated by density gradient centrifugation and cryopreserved. PBMC were thawed and stimulated with LPS (100 ng/ml) for 4 h and ATP (5 mM) for an additional hour. The cells were treated with metformin (5 mM) or MCC950 (100 nM) for 30 min prior to ATP addition. Monocytes were isolated by enriching PBMC for CD14^+^ cells (Miltenyi Biotec, Bergisch Gladbach, Germany) and resting overnight before treating with metformin (5 mM) or rapamycin (100 nM) for 30 min before stimulating with LPS (1 ng/ml) for 4 h.

**Explant cultures**

Skin biopsy thirds were cultured (~10 mg/ml) in Iscove’s Modified Dulbecco’s Medium (1% L-glutamine-penicillin-streptomycin, 0.5% human serum, Merck Life Sciences, Darmstadt Germany) and treated with metformin (5 mM) or AICAR (1 mM) or left untreated for 24 h. The explant conditioned media were assayed for secreted cytokines, while the skin was lysed for RNA isolation to quantify gene expression.

**Analysis of cytokine profiles**

Multiplex assays were carried out to quantify levels of CCL20, CXCL1, IFN-γ, IL-1α, IL-1β, IL-6, IL-8, IL-17A, IL-17AF, IL-17C, IL-18, IL-23, and TNF-α (Meso Scale Diagnostics, Rockville, MD) secreted from explants. In PBMC/monocyte cultures, IL-1β, IL-6 and TNF-α were measured by ELISA (BioLegend, San Diego, CA and Thermo Fisher Scientific, Waltham, MA).

**Analysis of gene expression**

RNA was extracted from PBMC or skin using a RNeasy Mini kit (Qiagen) and evaluated by Nanodrop (Thermo Fisher Scientific), then reverse transcribed using the high-capacity cDNA kit (Thermo Fisher Scientific). cDNA was analysed by quantitative RT-PCR using SYBR Green PCR Master Mix (Merck Life Sciences) for expression of *GLUT1*, *HK2*, *PFKFB3*, *IFN-γ*, *IL-17A*, *IL-6* and *TNF-α* (Microsynth, Wien, Austria) relative to the housekeeper gene ribosomal protein lateral stalk subunit P0 (*RPLP0*) (sequences in supplementary table 5). Relative quantitative data was obtained by normalising to the control.

**Characterising real-time metabolic profiles**

To examine their metabolic profile, PBMC (3x10^5^) were seeded in XFe96 microplates (Agilent Technologies, Santa Clara, CA) on Cell-Tak-coated wells (Corning, Corning, NY) and cultured in XF RPMI medium (1 mM HEPES, Agilent Technologies; 10 mM glucose, 2 mM L-glutamine and 1 mM pyruvate, Merck Life Sciences) and incubated for 1 h at 37°C (carbon dioxide-free). Baseline OCR and ECAR were measured before and following injection with oligomycin (2 μg/ml), carbonyl cyanide-4-(trifluoromethoxy)phenylhydrazone (FCCP, 5 μM), antimycin-A (2 μM)/rotenone (500 nM) and 2-deoxyglucose (2DG, 25 mM) (Merck Life Sciences) using the Seahorse XFe96 analyzer (Agilent Technologies). To measure the metabolic profile of skin explants, skin was dissected (10-20 mg pieces) and placed inside XFe24 islet capture microplates (Agilent Technologies) for 2 h at 37°C (carbon dioxide-free). Baseline OCR and ECAR values were measured using a Seahorse XFe24 analyzer, then metformin (5 mM) was added for 12 h, before repeating OCR and ECAR measurements.

**Flow cytometric analysis**

Monocytes were labelled with viability dye eFluor506 (Thermo Fisher Scientific) and fluorochrome-conjugated antibodies specific for CD14 (BV786, Becton Dickinson, Franklin Lakes, NJ) and CD64 (BV421, Becton Dickinson). The cells were fixed and permeabilised (Fix & Perm kit, Thermo Fisher Scientific) for intracellular staining using PS6 (AF488, Becton Dickinson). Fc receptor block was used to minimise non-specific antibody staining (Biolegend). The cells were acquired on a Cytek Aurora flow cytometer (Cytek Biosciences, Fremont, CA) and analysed using FlowJo software (FlowJo, Ashland, OR).
