# Supplementary material for: Metformin has anti-inflammatory effects and induces immunometabolic reprogramming via multiple mechanisms in hidradenitis suppurativa
Source: Br J Dermatol. 2023 Aug 30;189(6):730–40. doi: 10.1093/bjd/ljad305 (PMC13077222; doi:10.1093/bjd/ljad305)
Supplement: ljad305_Supplementary_Data [file ljad305_supplementary_data.zip › Figures S1–S4.docx]

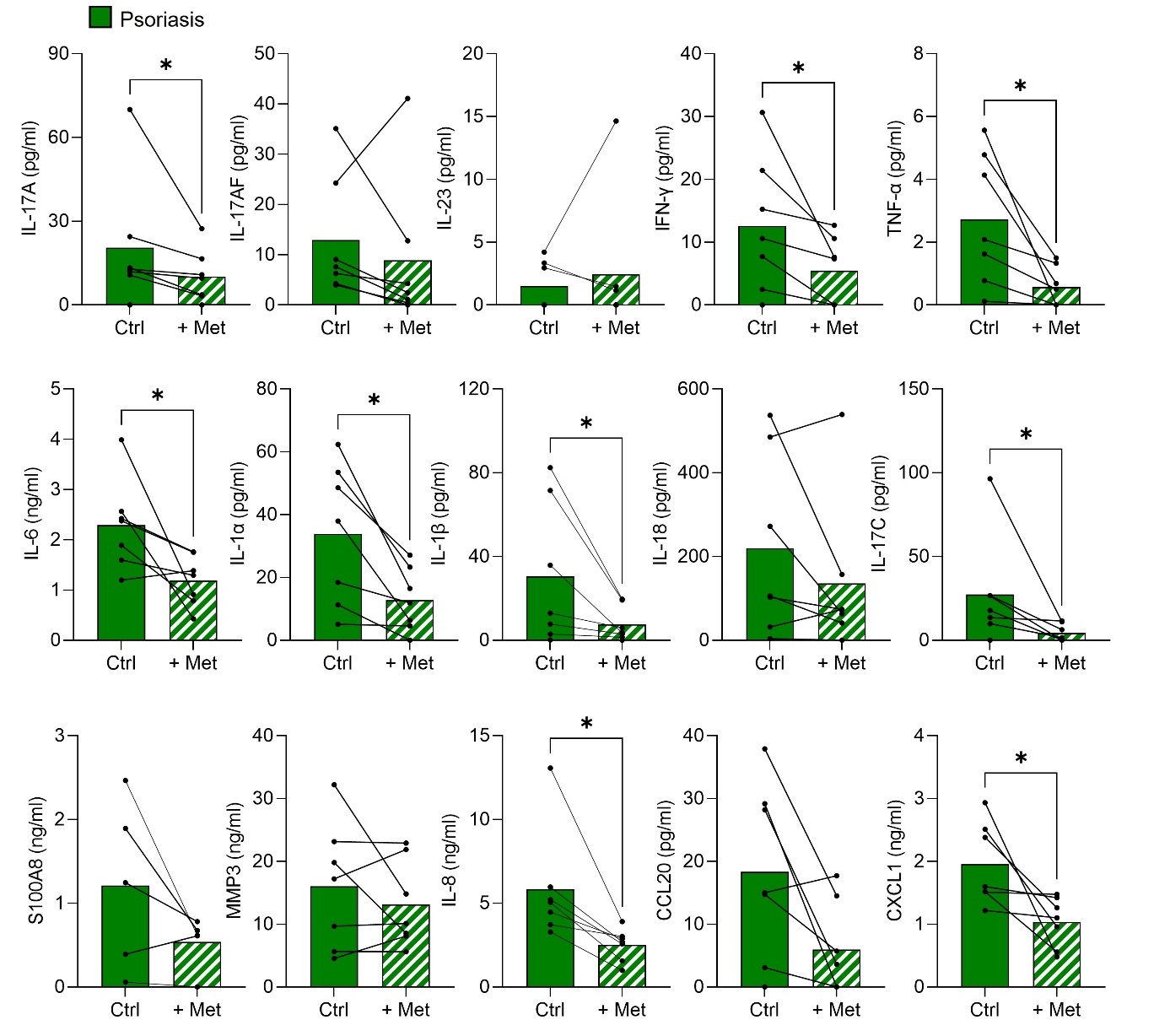


**Figure S1. Ex vivo metformin treatment reduces levels of pro-inflammatory cytokines and chemokines in psoriasis skin explants.** Explant cultures were set up using lesions from psoriasis patients (n=7) and cultured ± metformin (5 mM) for 24 h. Explant conditioned media were assayed for secretion of IL-17A, IL-17AF, IL-23, IFN-γ, TNF-α, IL-6, IL-1α, IL-1β, IL-18, IL-17C, S100A8, MMP-3, IL-8, CCL20 and CXCL1 by multiplex cytokine assay. Data are expressed as mean (± SEM) concentration. **P*<0.05 using Wilcoxon matched-pair signed rank tests.


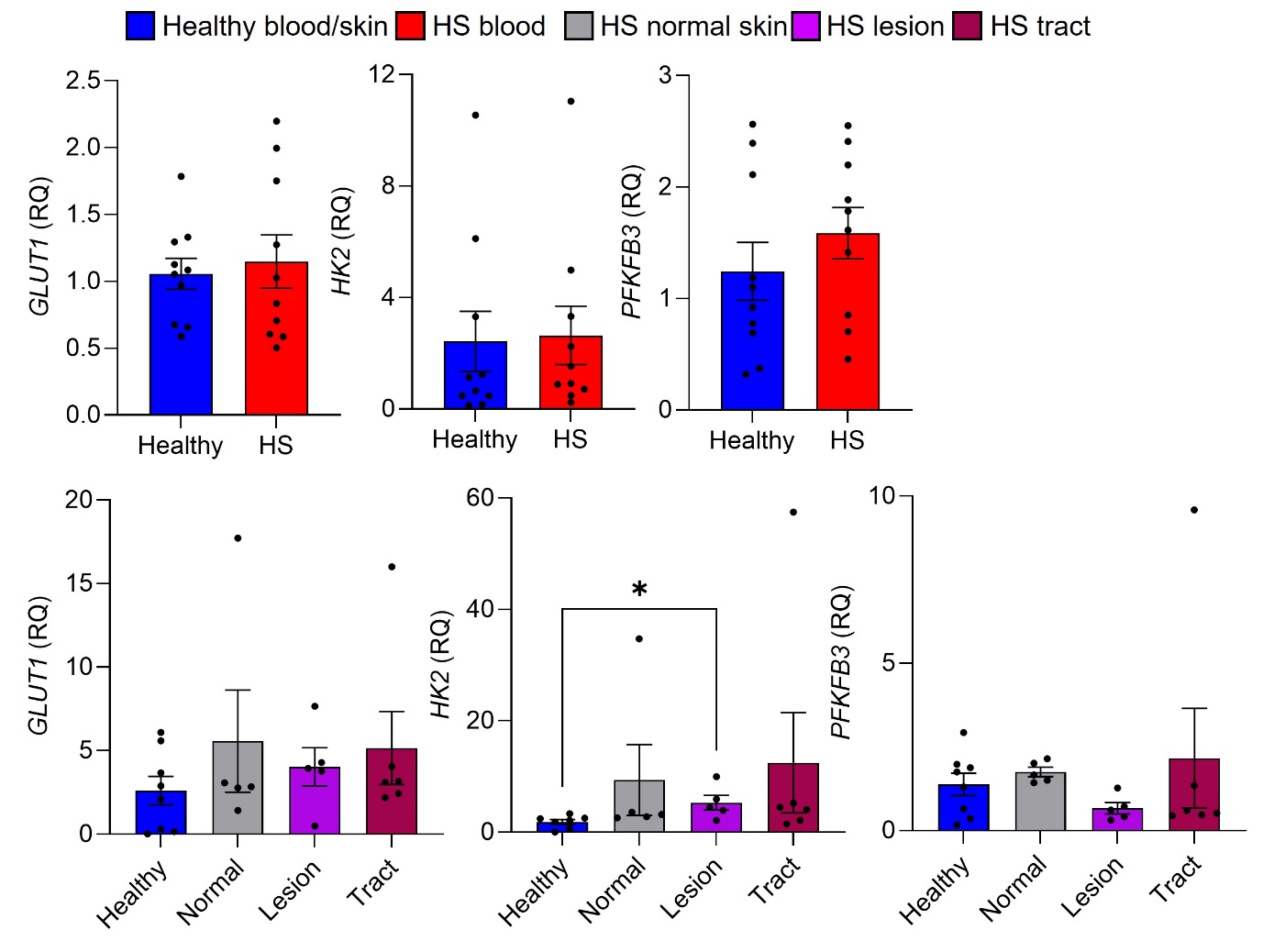


**Figure S2.** **Glycolytic markers are increased in skin of HS patients.** PBMC and skin biopsies from healthy donors (n=8-10) and HS patients (n=5-10) were lysed for RNA analysis. RNA was reverse transcribed to cDNA and analysed by quantitative RT-PCR for expression of *GLUT1*, *HK2* and *PFKFB3* relative to housekeeper gene *RPLP0.* Data are expressed as mean (± SEM) relative quantification (RQ). **P*<0.05 using Kruskal-Wallis test.


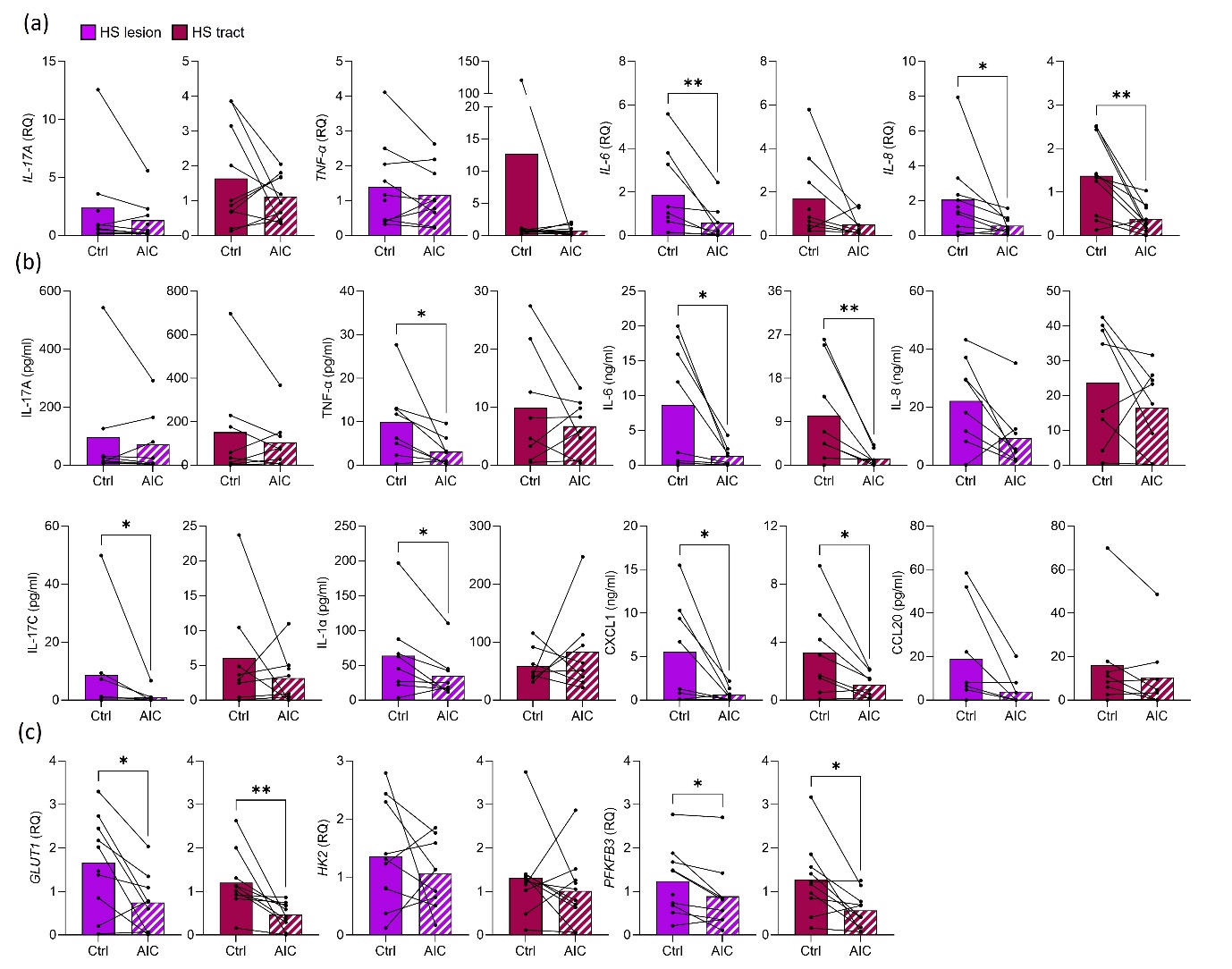


**Figure S3. Proinflammatory cytokines and chemokines are reduced by AICAR treatment ex vivo.** Explant cultures were set up using normal, lesion and tract samples from each HS patient (n=9-10) and cultured alone (Ctrl) or with AICAR (AIC) for 24 h. (a,c), the biopsies were lysed to isolate RNA, which was reverse transcribed to cDNA and analysed by quantitative RT-PCR for expression of *GLUT1*, *HK2* and *PFKFB3*, *IL-17A*, *TNF-α*, *IL-6*, *IL-8* relative to endogenous control gene *RPLP0*. (b), explant conditioned media were assayed for secretion of IL-17A, TNF-α, IL-6, IL-8, IL-17C, IL-1α, CXCL1 and CCL20 by multiplex cytokine assay. Data are expressed as mean (± SEM) relative quantification (RQ) or concentration. **P*<0.05, ***P*<0.01 using Wilcoxon matched-pair signed rank tests.


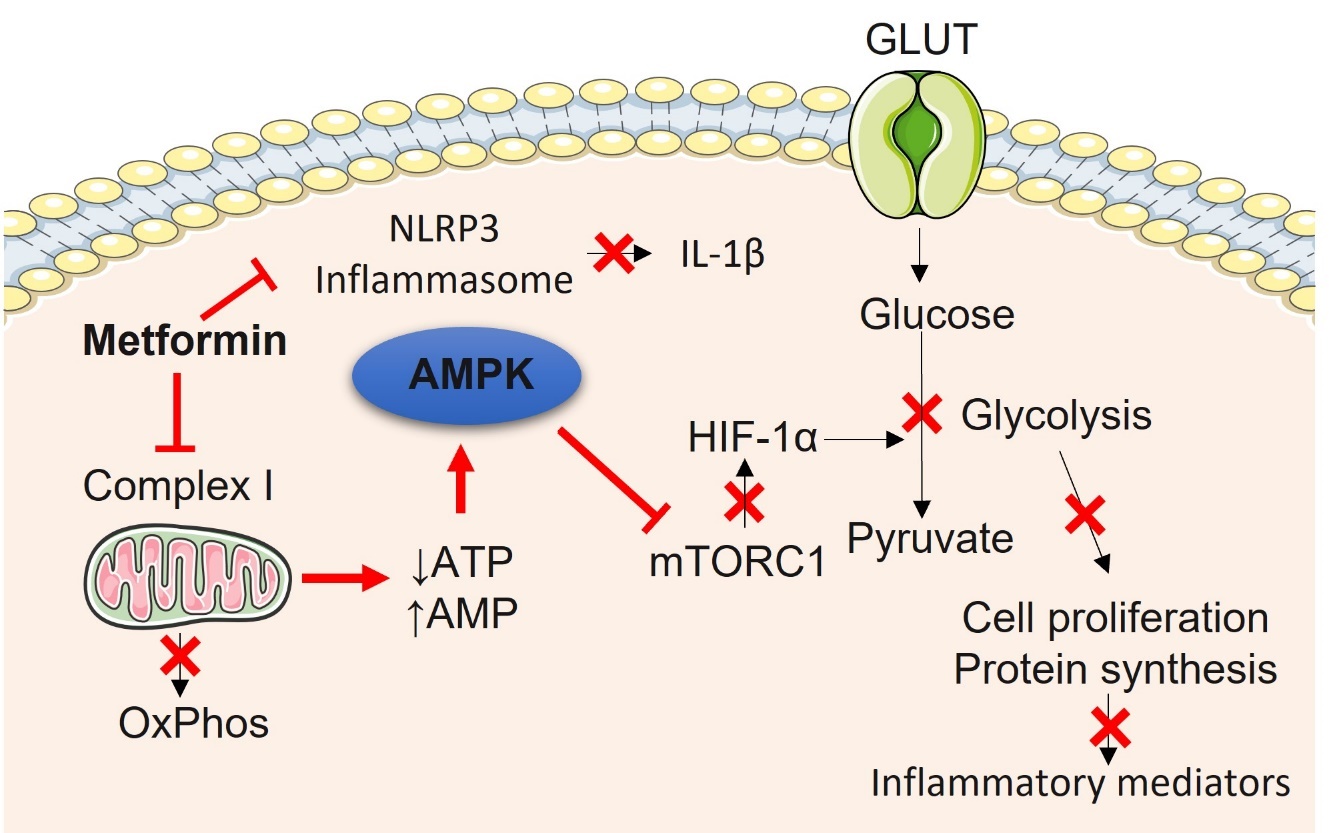


**Figure S4. Proposed mechanism of action of metformin in HS.** Metformin inhibits complex I of the mitochondrial electron transport chain, which suppresses OxPhos and boosts AMP levels, and indirectly activates AMPK. AMPK activation inhibits mTORC1, which can no longer activate HIF-1α, therefore inhibiting glycolysis. Glycolytic suppression inhibits cell proliferation and protein synthesis, thus preventing production of pro-inflammatory mediators. Metformin also inhibits the NLRP3 inflammasome, which is responsible for IL-1β production. AMP, adenosine monophosphate; AMPK, AMP-activated protein kinase; ATP, adenosine triphosphate; GLUT, glucose transporter; HIF, hypoxia inducible factor; mTORC, mechanistic target of rapamycin complex; NLRP3, NLR Family Pyrin Domain Containing 3; OxPhos, oxidative phosphorylation.
