# Supplementary material for: Metformin has anti-inflammatory effects and induces immunometabolic reprogramming via multiple mechanisms in hidradenitis suppurativa
Source: Br J Dermatol. 2023 Aug 30;189(6):730–40. doi: 10.1093/bjd/ljad305 (PMC13077222; doi:10.1093/bjd/ljad305)
Supplement: ljad305_Supplementary_Data [file ljad305_supplementary_data.zip › ljad305 JC.pptx]

## Slide 1
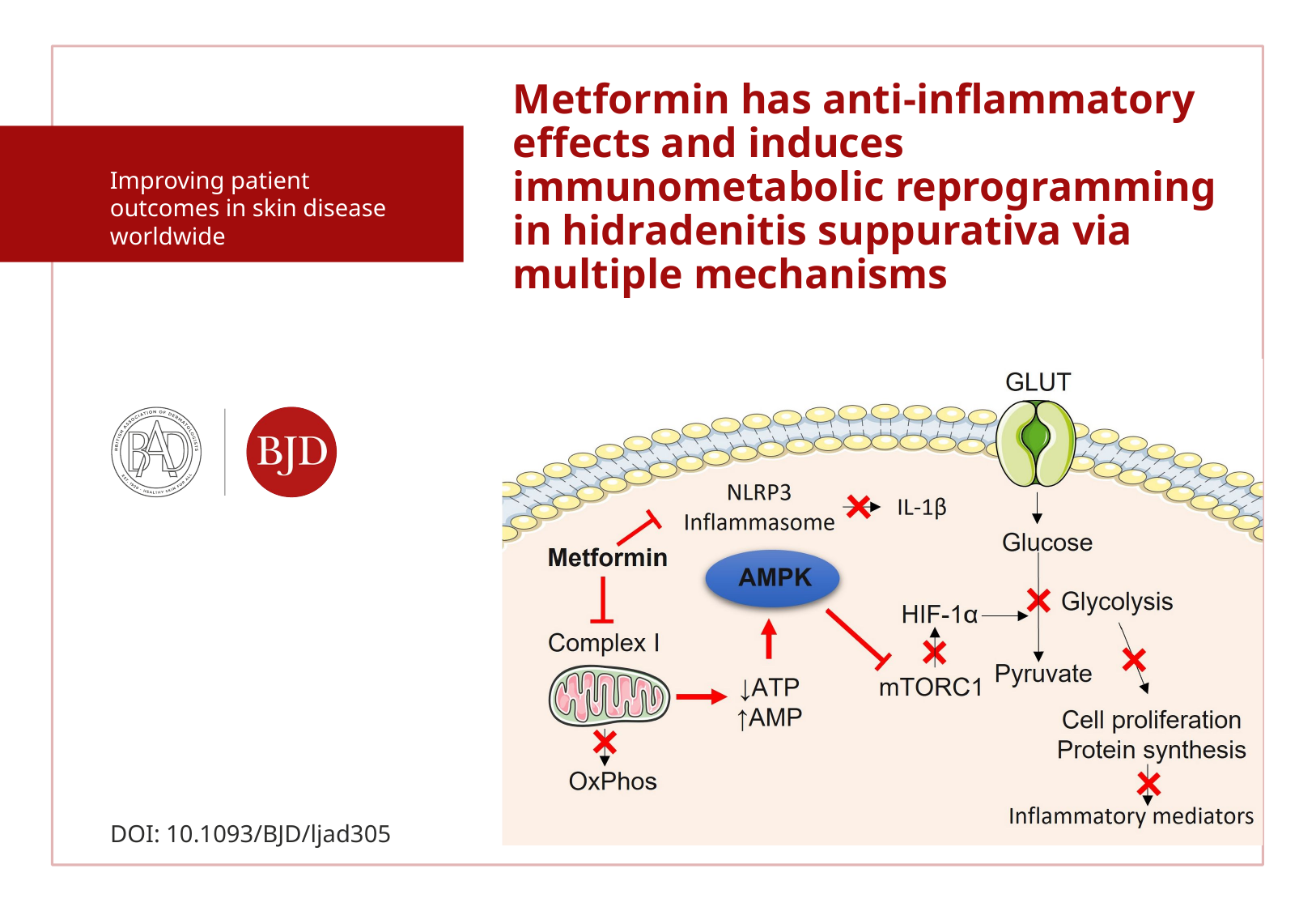

# Metformin has anti-inflammatory effects and induces immunometabolic reprogramming in hidradenitis suppurativa via multiple mechanisms
DOI: 10.1093/BJD/ljad305

## Slide 2
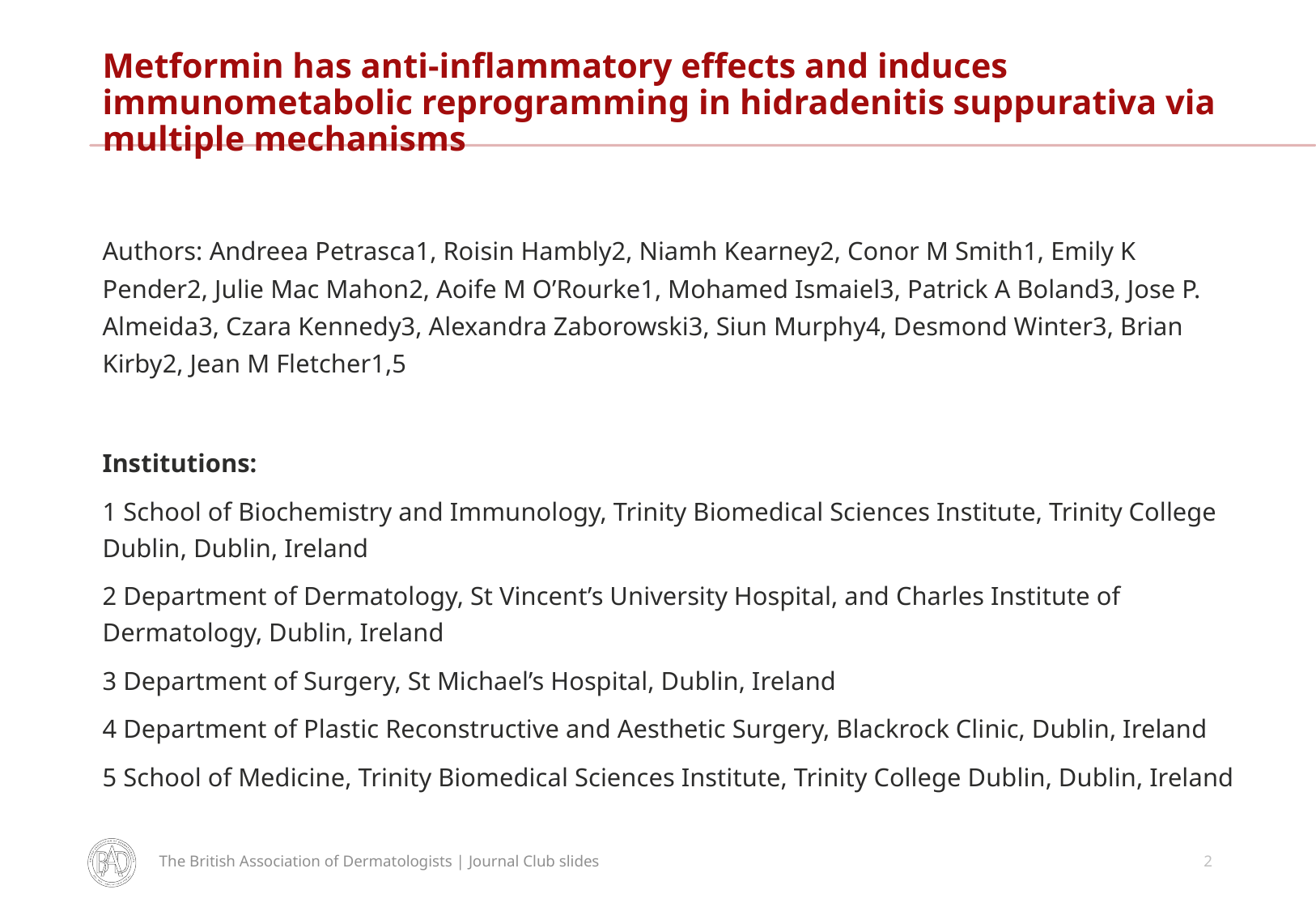

# Metformin has anti-inflammatory effects and induces immunometabolic reprogramming in hidradenitis suppurativa via multiple mechanisms
Authors: Andreea Petrasca1, Roisin Hambly2, Niamh Kearney2, Conor M Smith1, Emily K Pender2, Julie Mac Mahon2, Aoife M O’Rourke1, Mohamed Ismaiel3, Patrick A Boland3, Jose P. Almeida3, Czara Kennedy3, Alexandra Zaborowski3, Siun Murphy4, Desmond Winter3, Brian Kirby2, Jean M Fletcher1,5
Institutions:
1 School of Biochemistry and Immunology, Trinity Biomedical Sciences Institute, Trinity College Dublin, Dublin, Ireland
2 Department of Dermatology, St Vincent’s University Hospital, and Charles Institute of Dermatology, Dublin, Ireland
3 Department of Surgery, St Michael’s Hospital, Dublin, Ireland
4 Department of Plastic Reconstructive and Aesthetic Surgery, Blackrock Clinic, Dublin, Ireland
5 School of Medicine, Trinity Biomedical Sciences Institute, Trinity College Dublin, Dublin, Ireland
The British Association of Dermatologists | Journal Club slides
2

## Slide 3
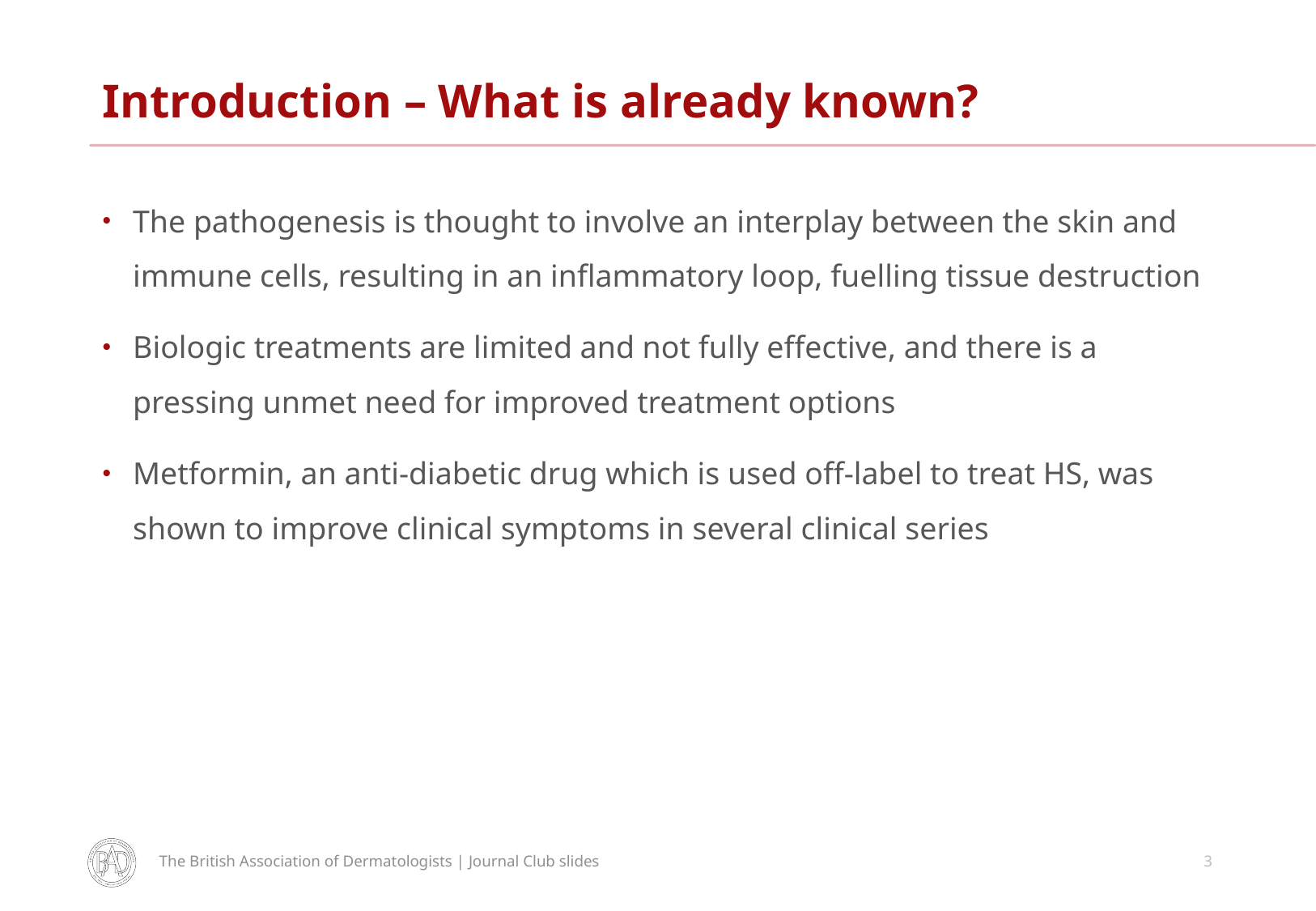

# Introduction – What is already known?
The pathogenesis is thought to involve an interplay between the skin and immune cells, resulting in an inflammatory loop, fuelling tissue destruction
Biologic treatments are limited and not fully effective, and there is a pressing unmet need for improved treatment options
Metformin, an anti-diabetic drug which is used off-label to treat HS, was shown to improve clinical symptoms in several clinical series
The British Association of Dermatologists | Journal Club slides
3

## Slide 4
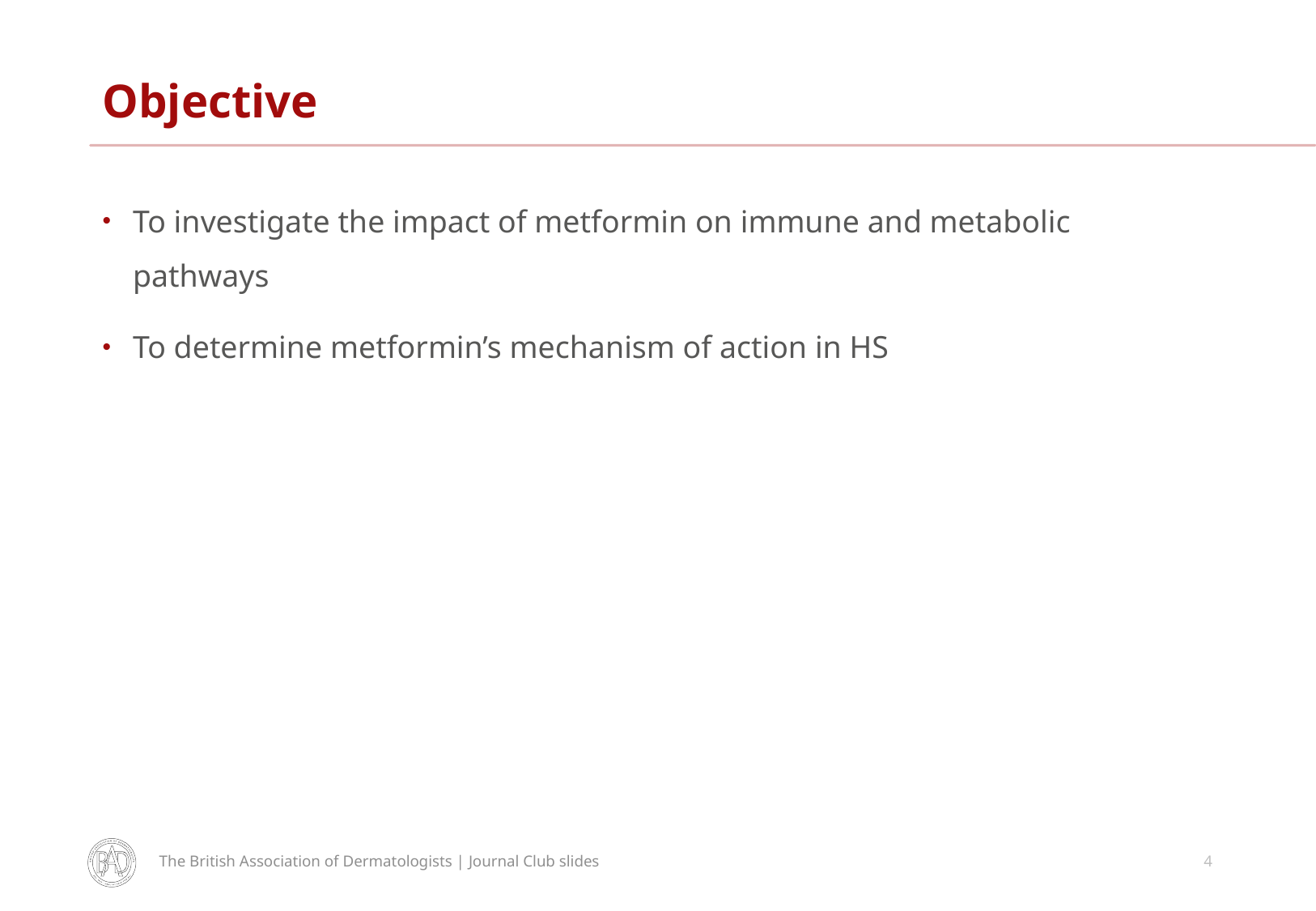

# Objective
To investigate the impact of metformin on immune and metabolic pathways
To determine metformin’s mechanism of action in HS
The British Association of Dermatologists | Journal Club slides
4

## Slide 5
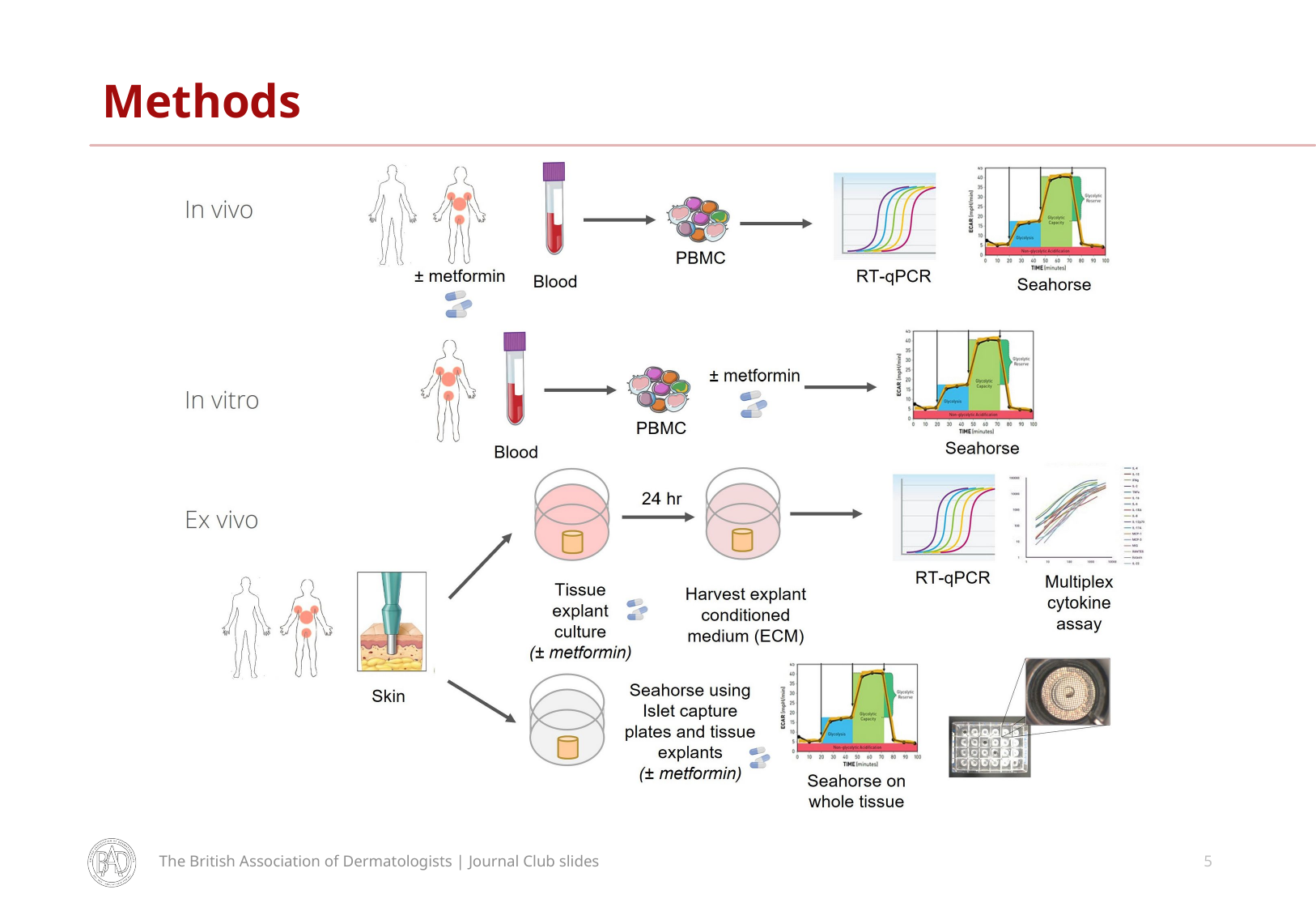

# Methods
The British Association of Dermatologists | Journal Club slides
5

## Slide 6
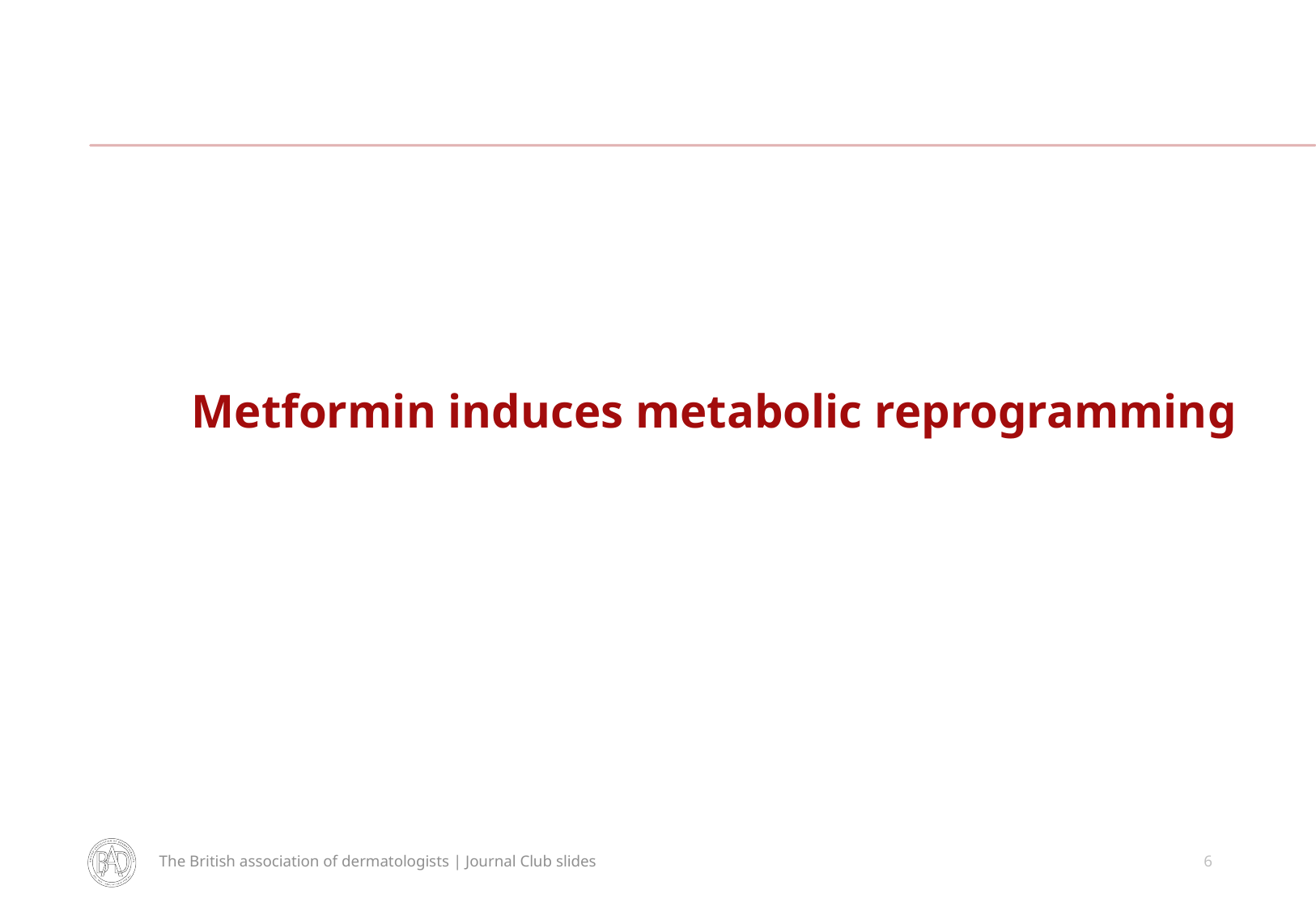

# Metformin induces metabolic reprogramming
The British association of dermatologists | Journal Club slides
6

## Slide 7
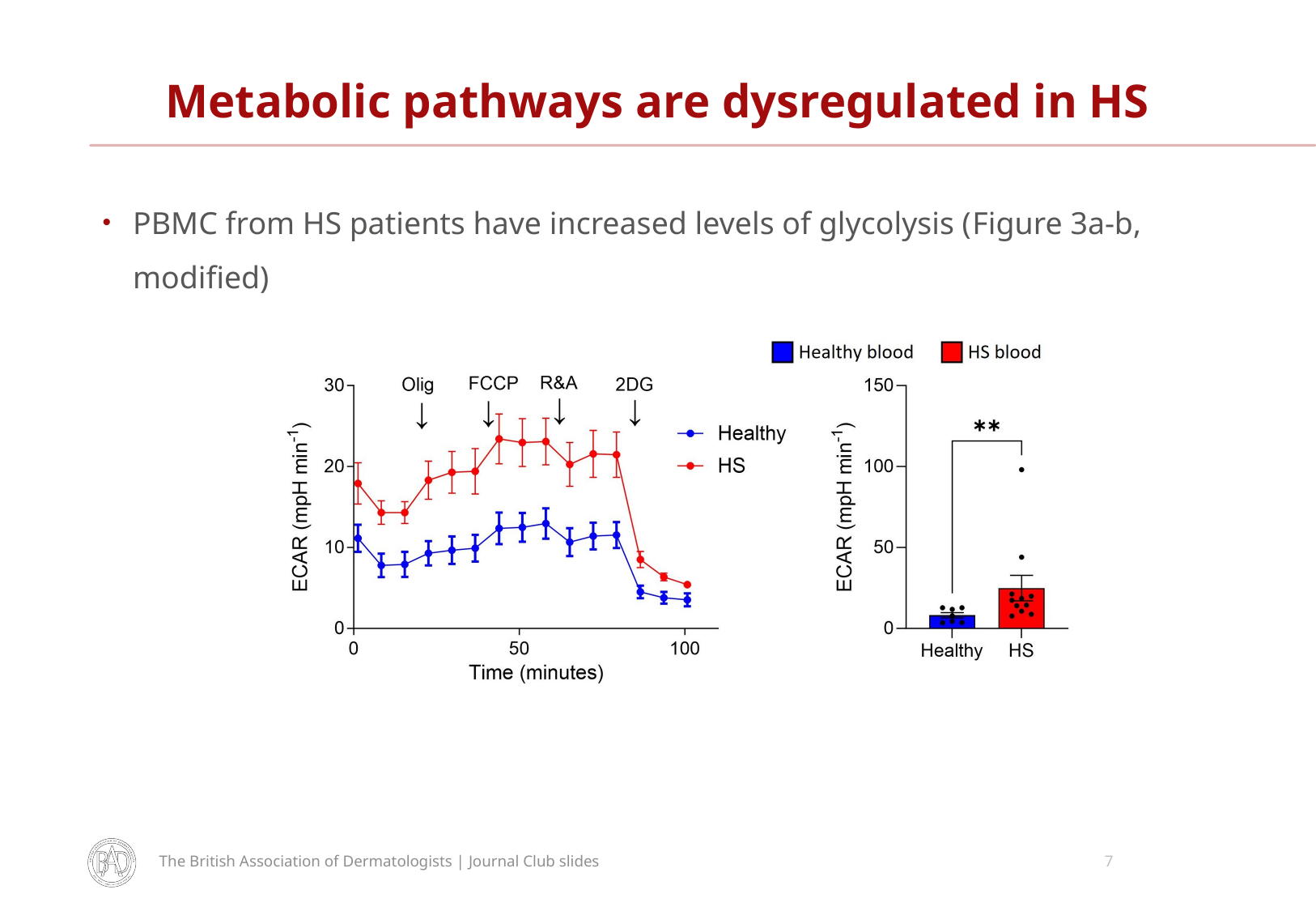

# Metabolic pathways are dysregulated in HS
PBMC from HS patients have increased levels of glycolysis (Figure 3a-b, modified)
The British Association of Dermatologists | Journal Club slides
7

## Slide 8
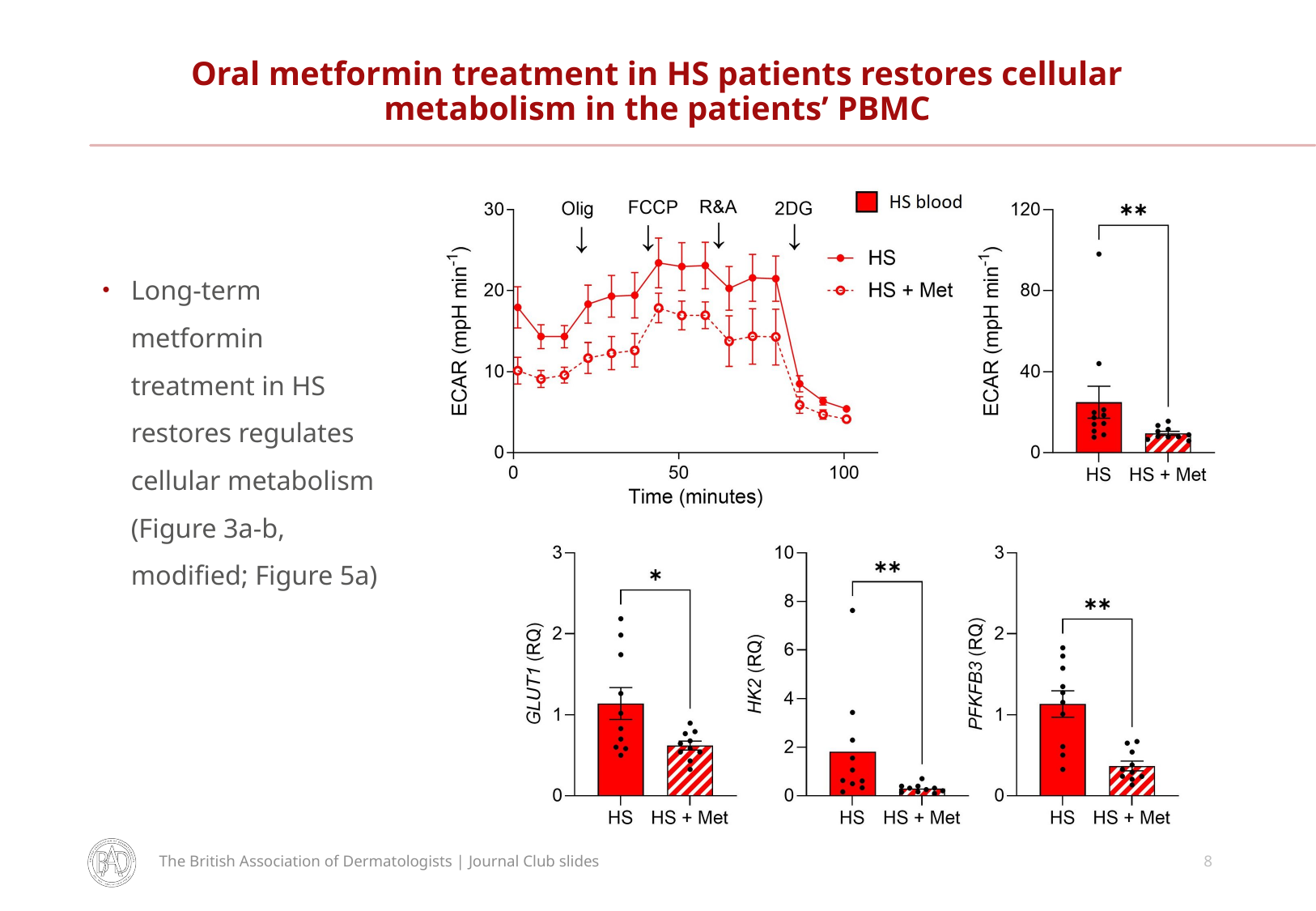

# Oral metformin treatment in HS patients restores cellular metabolism in the patients’ PBMC
Long-term metformin treatment in HS restores regulates cellular metabolism (Figure 3a-b, modified; Figure 5a)
The British Association of Dermatologists | Journal Club slides
8

## Slide 9
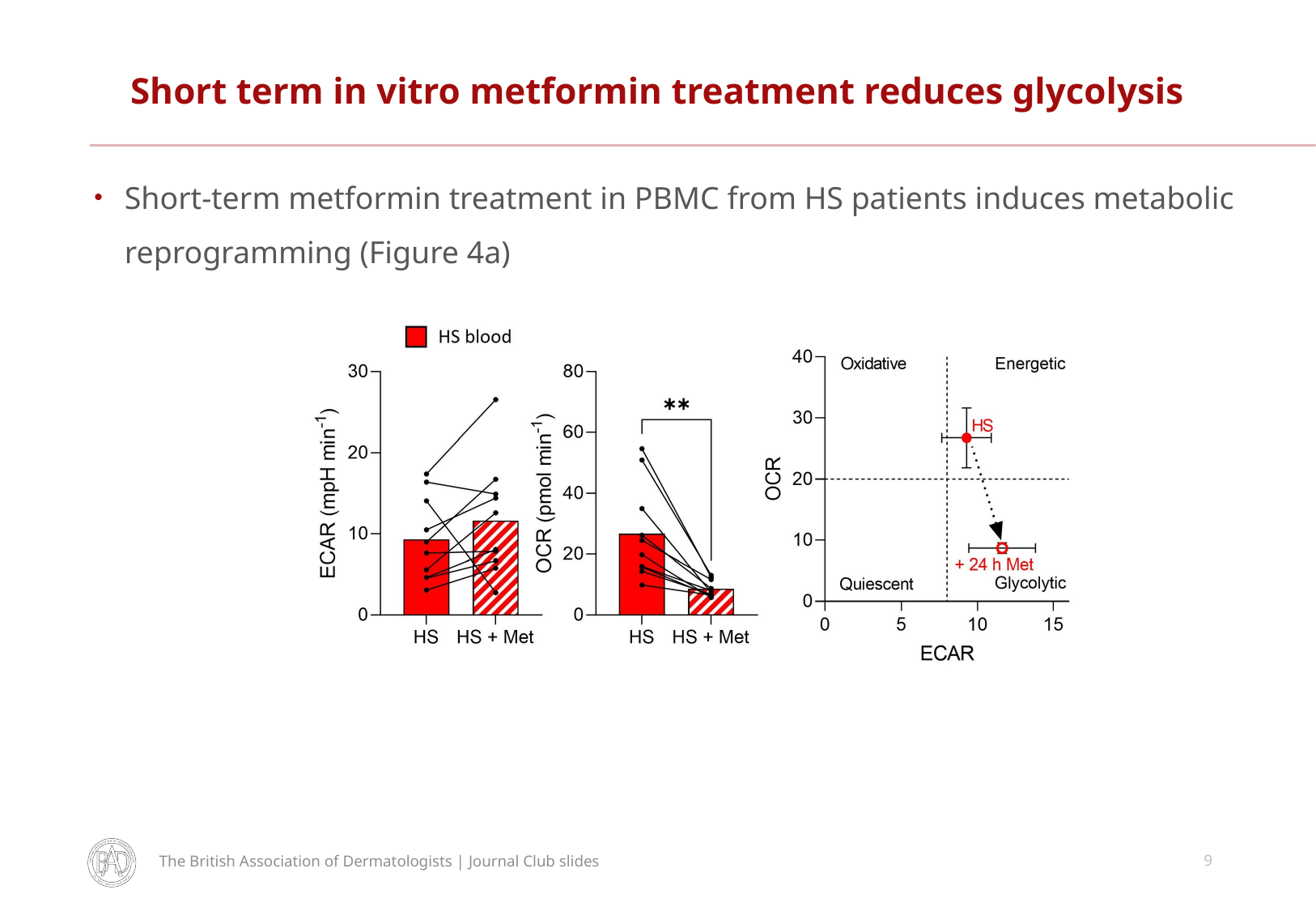

# Short term in vitro metformin treatment reduces glycolysis
Short-term metformin treatment in PBMC from HS patients induces metabolic reprogramming (Figure 4a)
The British Association of Dermatologists | Journal Club slides
9

## Slide 10
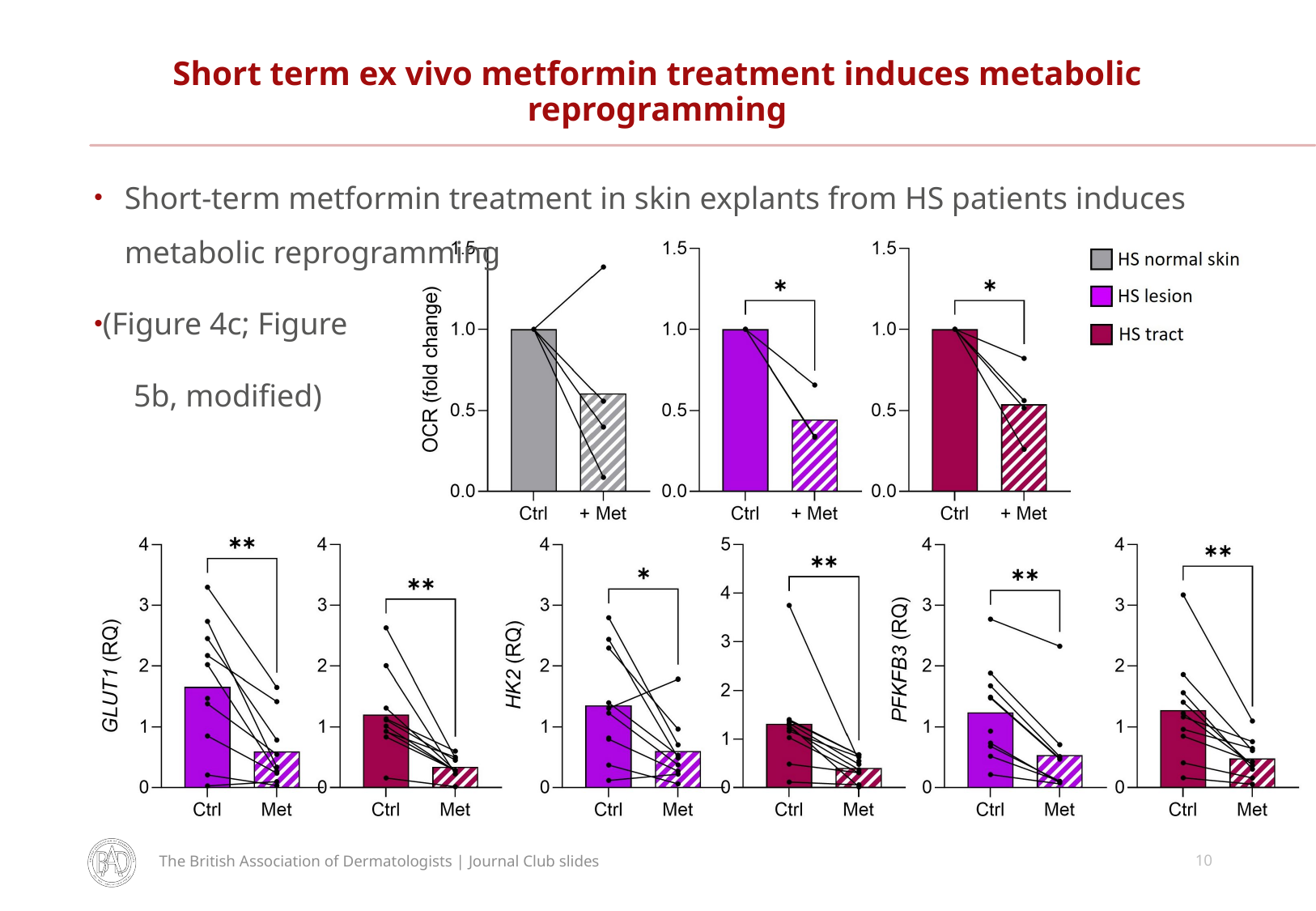

# Short term ex vivo metformin treatment induces metabolic reprogramming
Short-term metformin treatment in skin explants from HS patients induces metabolic reprogramming
(Figure 4c; Figure
 5b, modified)
The British Association of Dermatologists | Journal Club slides
10

## Slide 11
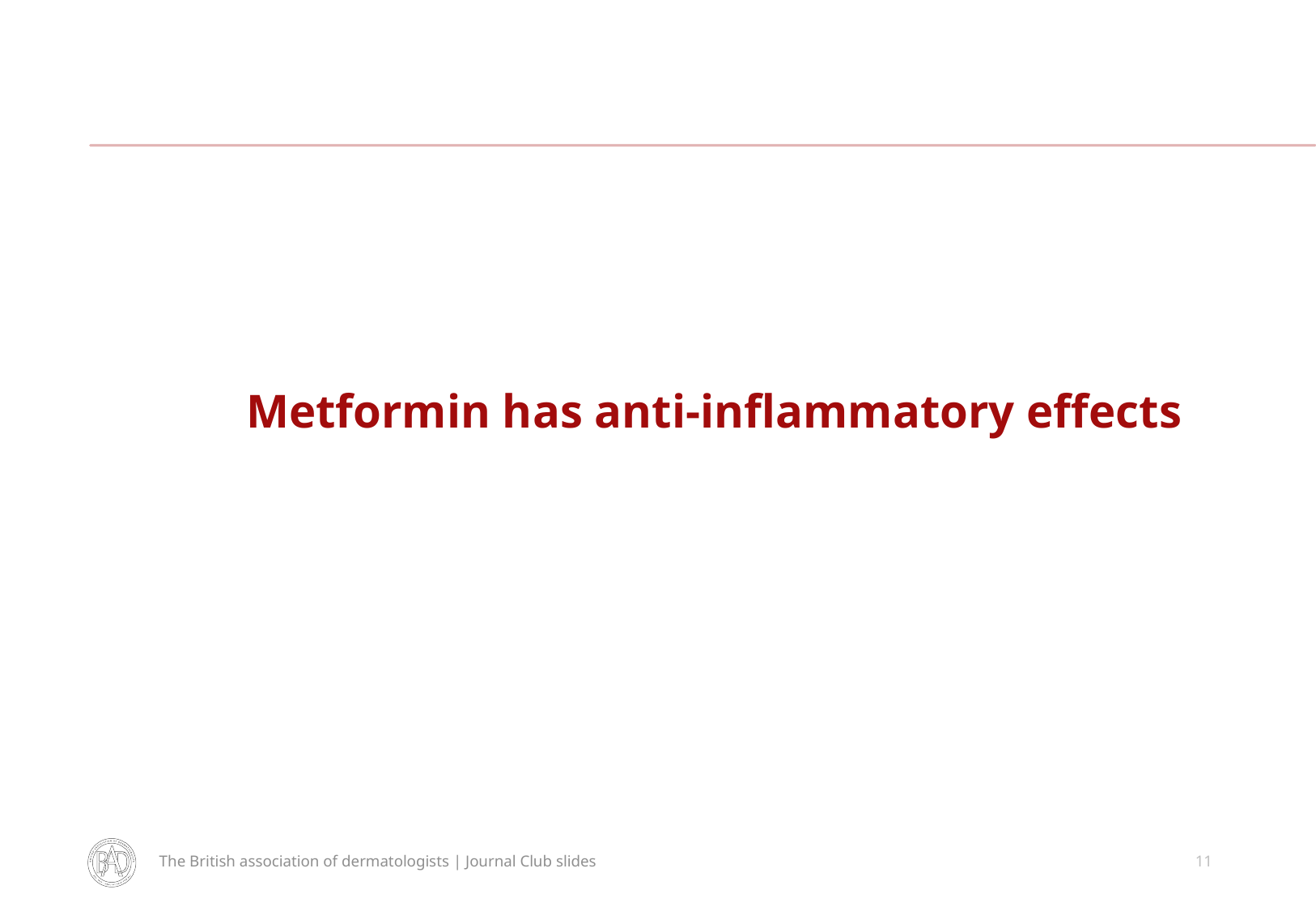

# Metformin has anti-inflammatory effects
The British association of dermatologists | Journal Club slides
11

## Slide 12
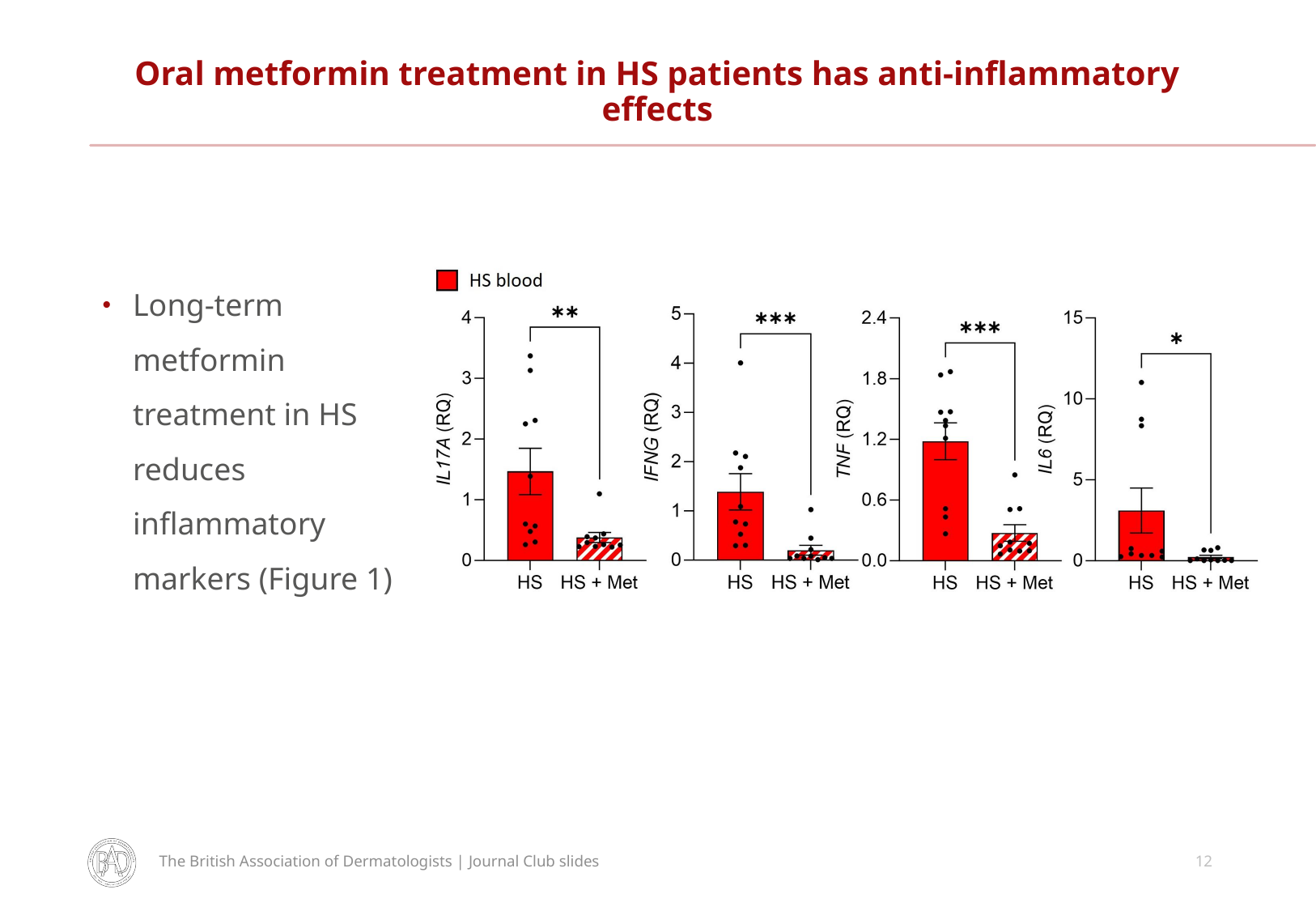

# Oral metformin treatment in HS patients has anti-inflammatory effects
Long-term metformin treatment in HS reduces inflammatory markers (Figure 1)
The British Association of Dermatologists | Journal Club slides
12

## Slide 13
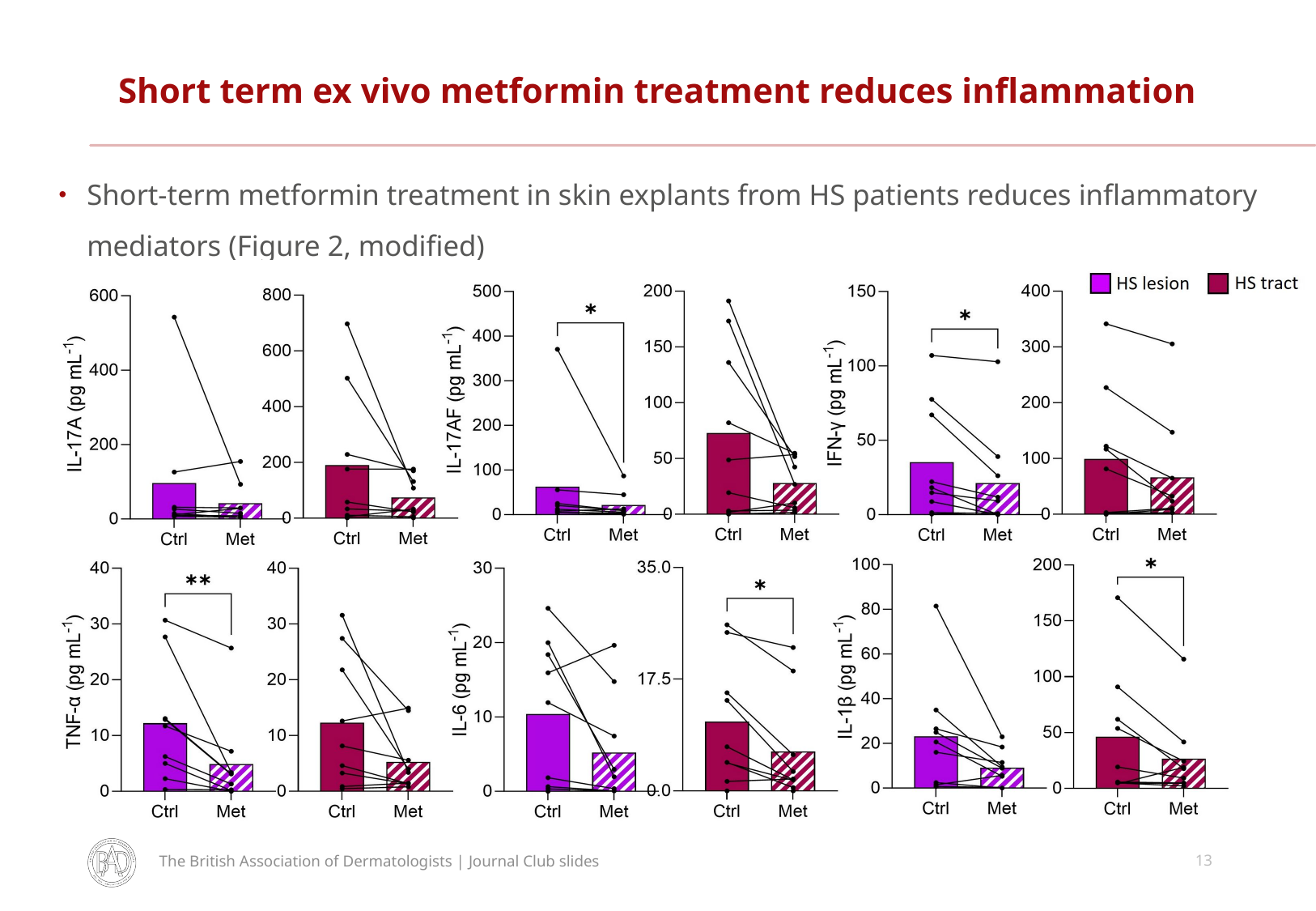

# Short term ex vivo metformin treatment reduces inflammation
Short-term metformin treatment in skin explants from HS patients reduces inflammatory mediators (Figure 2, modified)
The British Association of Dermatologists | Journal Club slides
13

## Slide 14
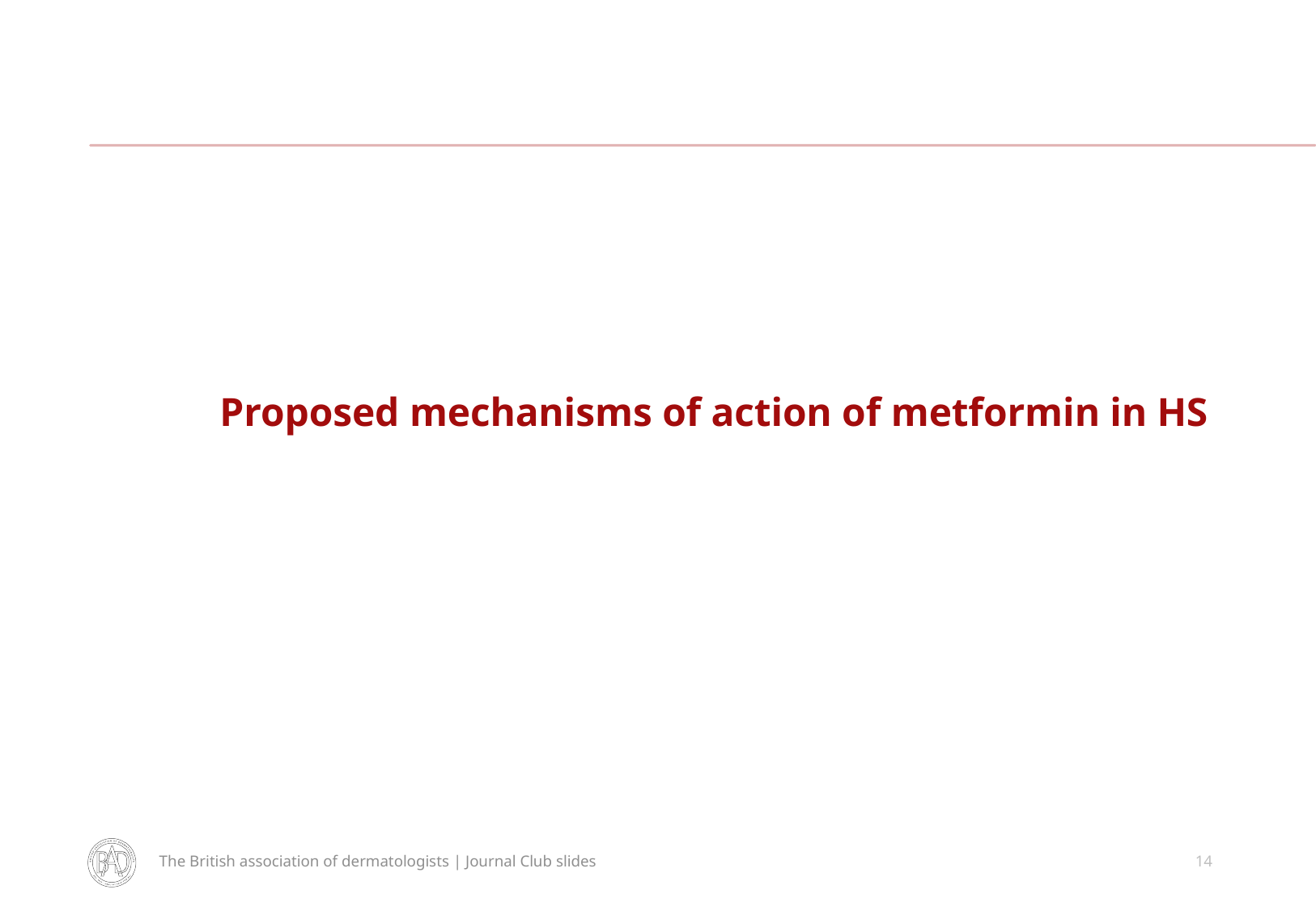

# Proposed mechanisms of action of metformin in HS
The British association of dermatologists | Journal Club slides
14

## Slide 15
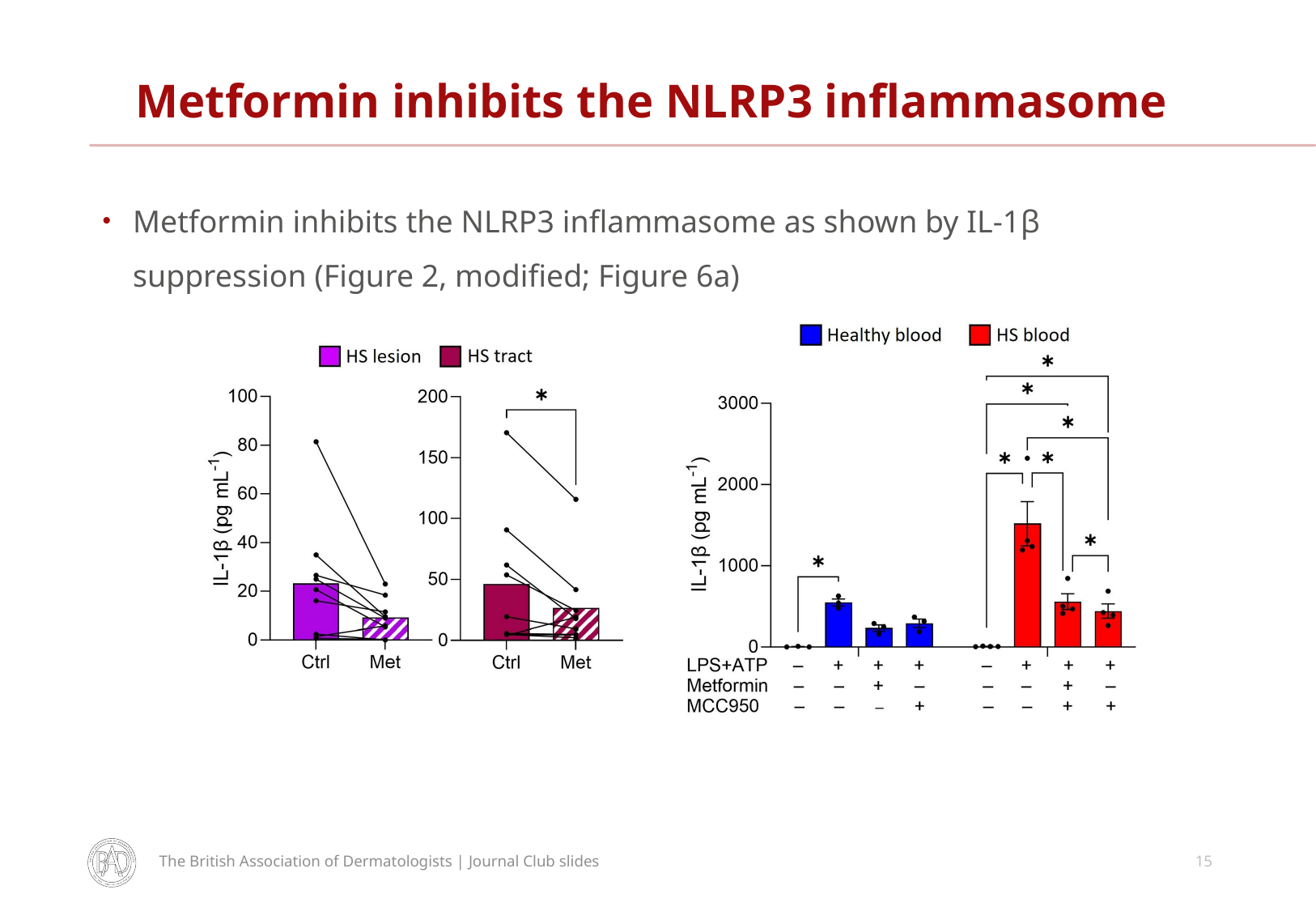

# Metformin inhibits the NLRP3 inflammasome
Metformin inhibits the NLRP3 inflammasome as shown by IL-1β suppression (Figure 2, modified; Figure 6a)
The British Association of Dermatologists | Journal Club slides
15

## Slide 16
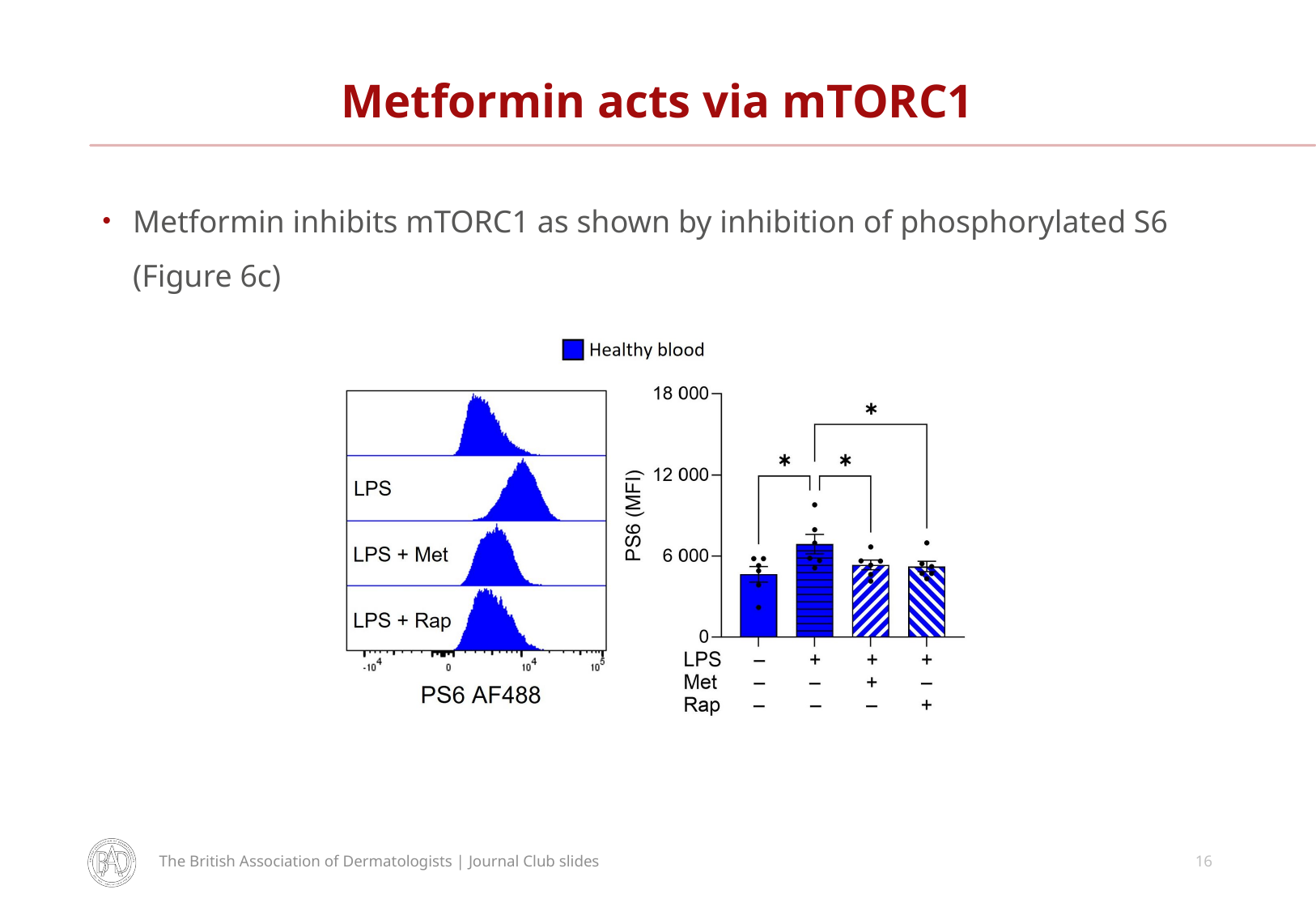

# Metformin acts via mTORC1
Metformin inhibits mTORC1 as shown by inhibition of phosphorylated S6 (Figure 6c)
The British Association of Dermatologists | Journal Club slides
16

## Slide 17
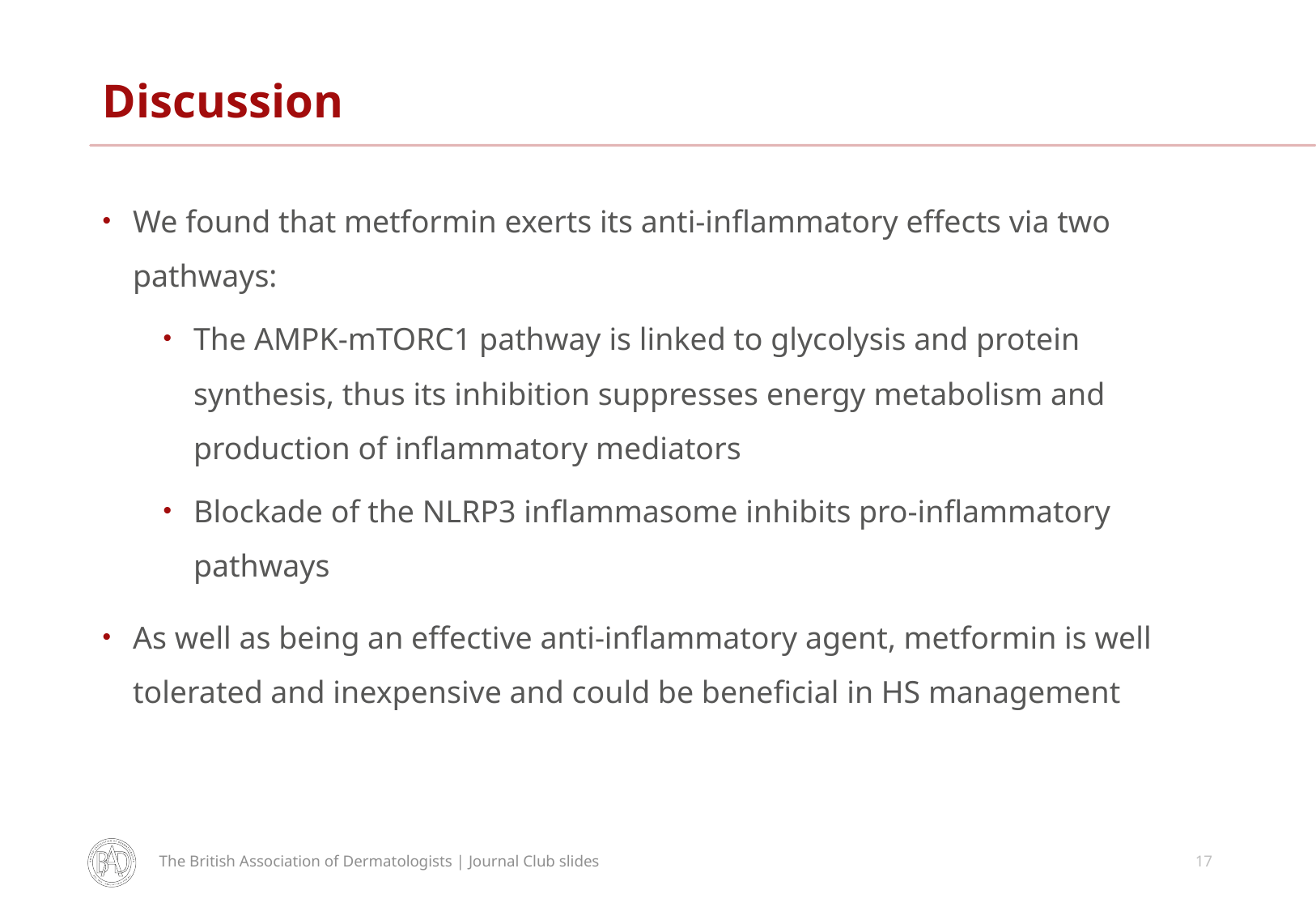

# Discussion
We found that metformin exerts its anti-inflammatory effects via two pathways:
The AMPK-mTORC1 pathway is linked to glycolysis and protein synthesis, thus its inhibition suppresses energy metabolism and production of inflammatory mediators
Blockade of the NLRP3 inflammasome inhibits pro-inflammatory pathways
As well as being an effective anti-inflammatory agent, metformin is well tolerated and inexpensive and could be beneficial in HS management
The British Association of Dermatologists | Journal Club slides
17

## Slide 18
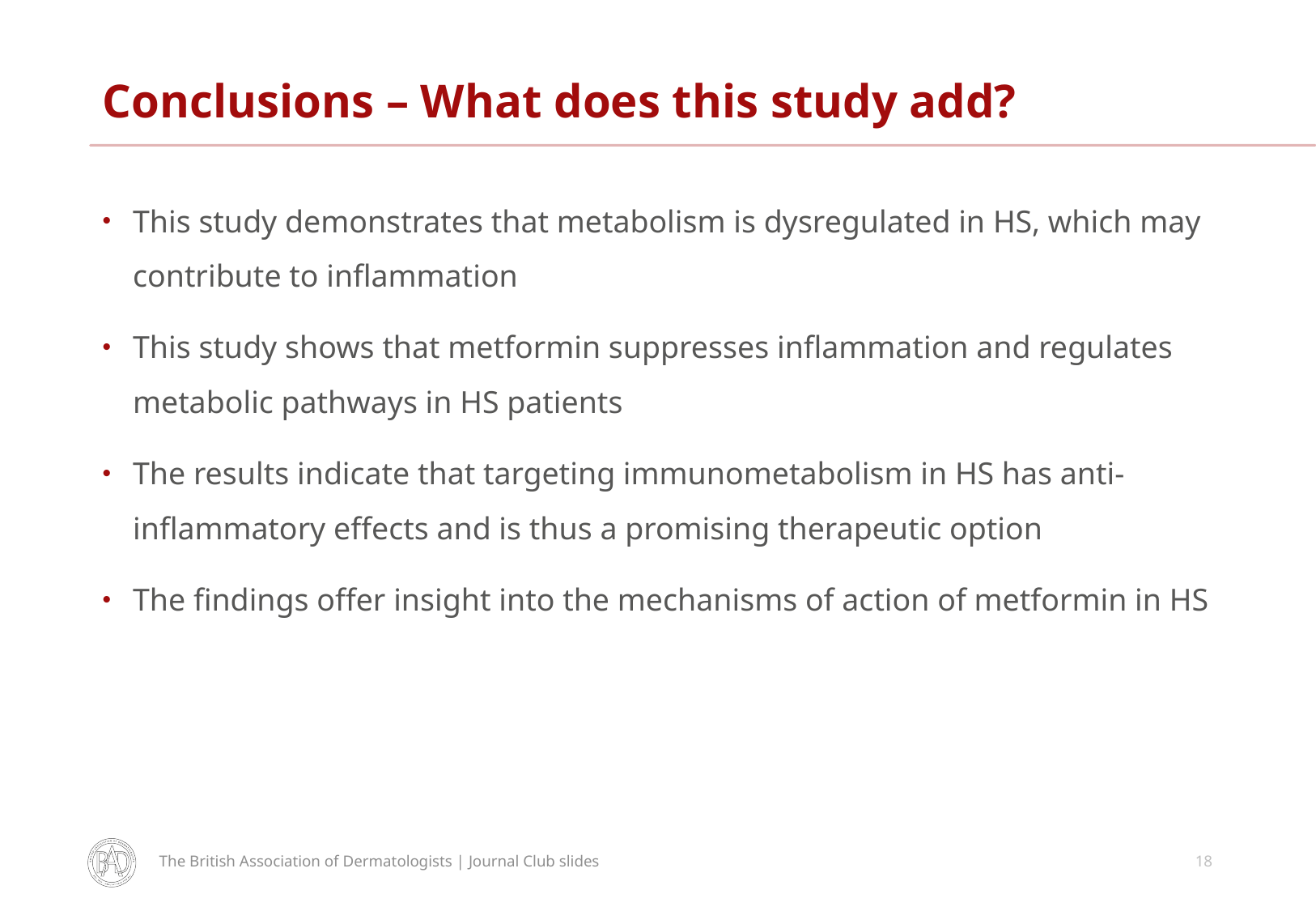

# Conclusions – What does this study add?
This study demonstrates that metabolism is dysregulated in HS, which may contribute to inflammation
This study shows that metformin suppresses inflammation and regulates metabolic pathways in HS patients
The results indicate that targeting immunometabolism in HS has anti-inflammatory effects and is thus a promising therapeutic option
The findings offer insight into the mechanisms of action of metformin in HS
The British Association of Dermatologists | Journal Club slides
18

## Slide 19
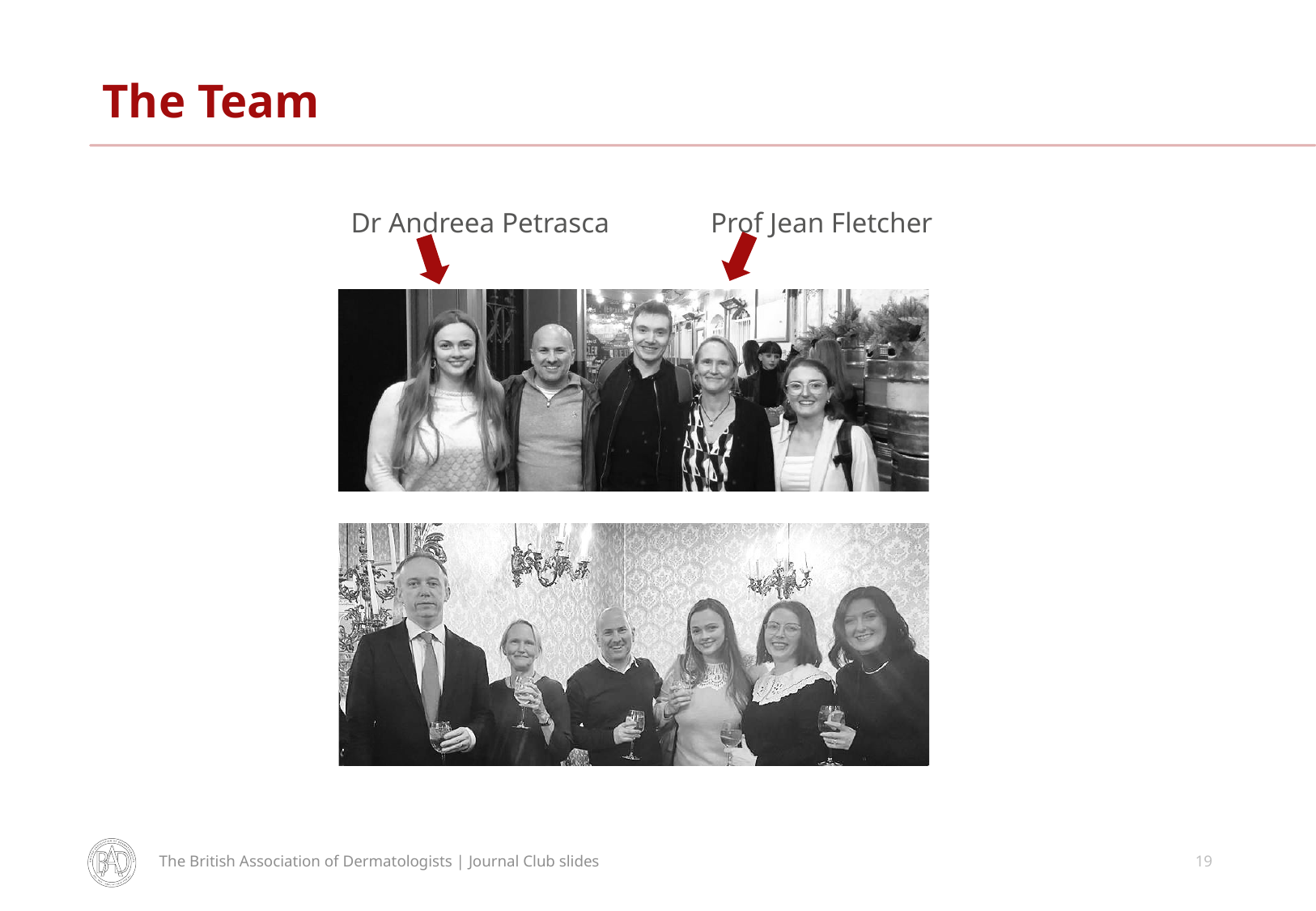

# The Team
Prof Jean Fletcher
Dr Andreea Petrasca
The British Association of Dermatologists | Journal Club slides
19

## Slide 20
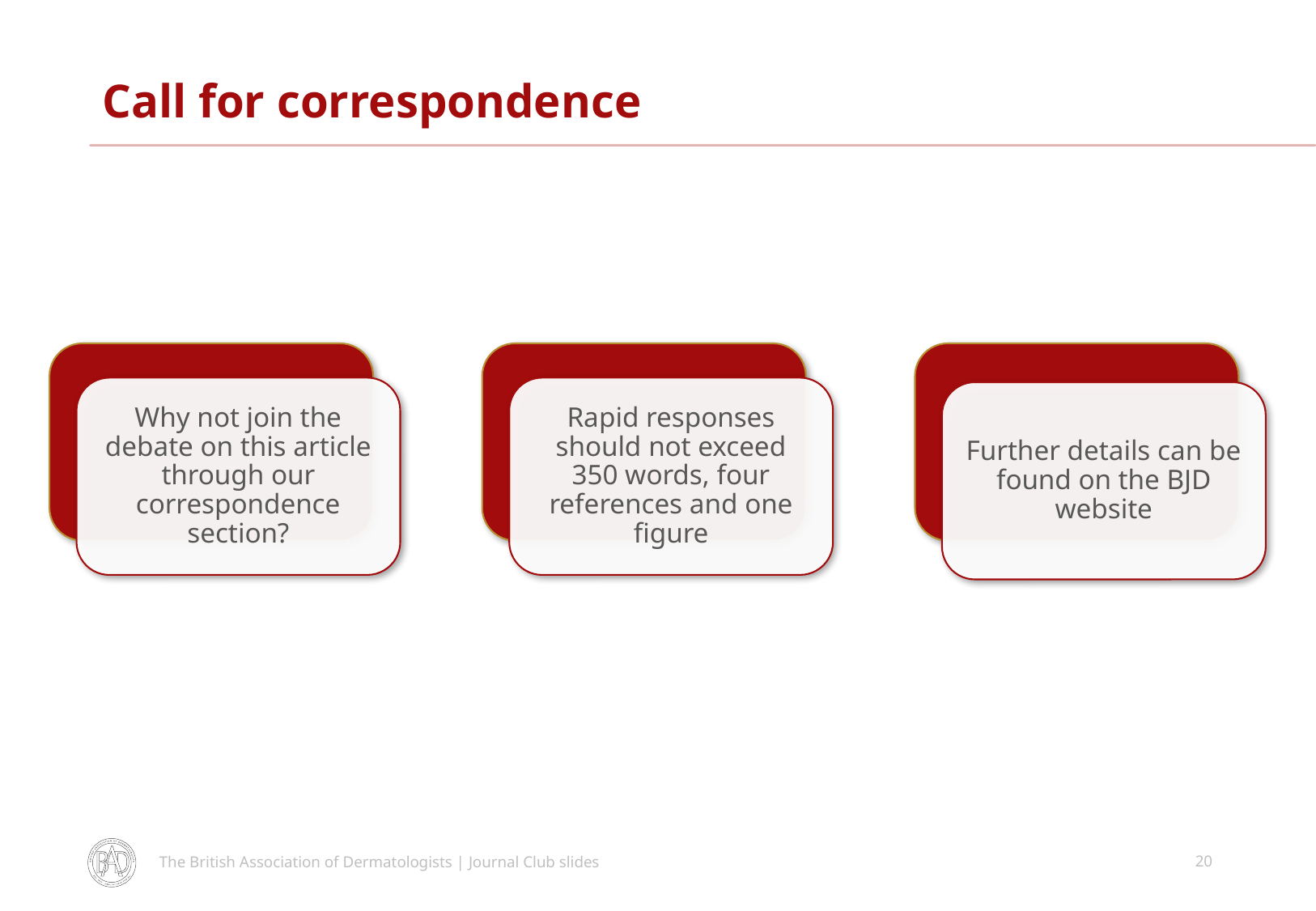

# Call for correspondence
Why not join the debate on this article through our correspondence section?
Rapid responses should not exceed 350 words, four references and one figure
Further details can be found on the BJD website
The British Association of Dermatologists | Journal Club slides
20
