# Supplementary material for: Metformin has anti-inflammatory effects and induces immunometabolic reprogramming via multiple mechanisms in hidradenitis suppurativa
Source: Br J Dermatol. 2023 Aug 30;189(6):730–40. doi: 10.1093/bjd/ljad305 (PMC13077222; doi:10.1093/bjd/ljad305)
Supplement: ljad305_Supplementary_Data [file ljad305_supplementary_data.zip › Tables S1–S5.docx]

**Table S1.** Healthy controls

| ID | Age | Sex | BMI | Smoker | Sample type | Fig |
| --- | --- | --- | --- | --- | --- | --- |
| HC14 | 30 | F |  |  | Blood | 3 |
| HC17 | 24 | M | 21.6 | N | Blood | 3,S2 |
| HC26 | 29 | F | 24.2 | N | Blood | 3 |
| HC27 | 44 | M | 23.5 | N | Blood | 3 |
| HC28 | 38 | F | 20.5 |  | Blood | 3 |
| HC30 | 25 | M | 21.6 | N | Blood | 3,6 |
| HC31 | 31 | F | 29.1 | N | Blood | 3,S2 |
| HC40 | 23 | F | 30.5 | N | Blood | 6 |
| HC41 | 38 | M | 25.4 | N | Blood | 6 |
| HC42 | 37 | F | 25.8 | N | Blood | 6 |
| HC43 | 26 | M | 21.3 | N | Blood | 6 |
| HC44 | 46 | M | 23.5 | N | Blood | 6 |
| HC45 | 32 | F | 29.8 | N | Blood | 6 |
| hc50 | 31 | F | 21.5 | N | Blood | 6 |
| hc51 | 29 | F | 22.2 | N | Blood | 6 |
| HC52 | 34 | M | 25.4 | N | Blood | 6 |
| HC53 | 32 | M | 24.9 | N | Blood | 6 |
| HC18 | 45 | F |  | N | Blood | S2 |
| HC19 | 37 | M |  |  | Blood | S2 |
| HC20 | 50 | F |  |  | Blood | S2 |
| HC21 | 27 | F |  |  | Blood | S2 |
| HC22 | 31 | F |  |  | Blood | S2 |
| HC29 | 34 | M | 26.5 |  | Blood | S2 |
| HC32 | 32 | F | 23.6 | N | Blood | S2 |
| HC33 | 31 | F | 25 | N | Blood | S2 |
| SVPH6 | 44 | F |  |  | Surgical | S2 |
| SVPH8 |  | F |  |  | Surgical | S2 |
| SVPH9 |  | F |  |  | Surgical | S2 |
| BRC5 | 69 | F |  |  | Surgical | S2 |
| UCD01 | 63 | F |  |  | Surgical | S2 |
| UCD02 | 42 | F |  |  | Surgical | S2 |
| UCD03 | 60 | F |  |  | Surgical | S2 |
| UCD04 | 43 | F |  |  | Surgical | S2 |
| UCD05 |  |  |  |  | Surgical | S2 |
| UCD14 | 47 | F |  |  | Surgical | S2 |

**Table S2.** Hidradenitis suppurativa patients

| ID | Age (Y) | Sex | BMI | Hurley stage | Smo-  ker | Sample type | Medications | Comorbidities | Fig |
| --- | --- | --- | --- | --- | --- | --- | --- | --- | --- |
| BK03 | 29 | F | 44.3 |  | Y | Blood | Ustekinumab | Obesity | 1,5,S2 |
| BK05 | 35 | F |  |  | N | Blood | Clindamycin, rifampicin | Obesity | 1,5,S2 |
| BK07 | 52 | F |  |  | N | Blood | Infliximab IV | Nil | 1,5,S2 |
| BK41 | 30 | M | 33.1 |  | Ex | Blood | Dapsone | Nil | 1,5,S2 |
| BK42 | 39 | F | 46.5 |  | Y | Blood | Adalimumab | Nil | 1,5,S2 |
| BK43 | 42 | F | 29.8 |  | Y | Blood | Nil | Nil | 1,5,S2 |
| BK44 | 46 | M | 24.3 |  | Ex | Blood | Dapsone | Nil | 1,5,S2 |
| HSM23 | 23 | F | 56.1 | 2 | N | Blood | Sertraline, Mirena coil | Obesity, depression, anxiety, rheumatoid arthritis | 1,5,S2 |
| HSM24 | 26 | M | 26 | 2 | Y | Blood | Tetracycline | Nil | 1,5,S2 |
| HSM26 | 27 | F | 37.5 | 2 | Ex | Blood | Clindamycin, rifampicin, OCP | Nil | 1,5,S2 |
| HSM21 | 23 | F | 35.9 | 2 | N | Blood | Metformin, evra patch, spironolactone, neoclarytin, inhalers | Polycystic ovarian syndrome, seizure disorder, pityriasis lichenoides chronicus | 1,5 |
| HSM22 | 30 | F | 52.7 | 2 | N | Blood | Metformin | Arthritis | 1,5 |
| HSM25 | 60 | F | 33.6 | 2 | Y | Blood | Metformin, clopidogrel, coversyl, aspirin, elecon eltroxin, esomeprazole, daktacort, euromavet, atorvas, | Hypertension, hypothyroid, cerebrovascular accident | 1,5 |
| HSM36 | 48 | F | 44.9 | 2 | Y | Blood | Metformin | Nil | 1,5 |
| HSM37 | 22 | F | 28.1 | 2 | N | Blood | Metformin | Nil | 1,3,5 |
| HSM38 | 26 | F | 29 | 2 | N | Blood | Metformin | Nil | 1,5 |
| HSM39 | 41 | F | 55.6 | 2 | Y | Blood | Metformin | Polycystic ovarian syndrome, inflammatory bowel disease | 1,5 |
| HSM40 | 39 | F | 26.1 | 3 | Y | Blood | Metformin, lamotrigine | Bipolar disorder | 1,5 |
| HSM41 | 33 | F | 38.2 | 3 | Y | Blood | Metformin | Nil | 1,3,5 |
| HSM42 | 26 | F | 20.1 | 1 | N | Blood | Metformin, spironolactone | Nil | 1,5 |
| MI01 | 58 | F | 39.4 | 3 | Ex | Surgical | Adalimumab | Chronic obstructive pulmonary disease depression, hypercholesterolemia | 2,S3 |
| MI02 | 52 | F | 32.4 | 3 | Ex | Surgical | Adalimumab, infliximab | Fatty liver | 2,S3 |
| MI03 | 50 | F | 32.4 | 3 | Ex | Surgical | Certolizumab | Hypercholesterolemia, peptic ulcer | 2,S3 |
| MI04 | 26 | F | 48.4 | 2 | N | Surgical | Brodalumab, minocycline | Sleep apnoea, depression | 2,S3 |
| MI05 | 22 | F | 51 | 2 | Ex | Surgical | Nil | Nil | 2,S2,S3 |
| MI06 | 34 | F | 40.3 | 2 | Y | Surgical | Nil | Nil | 2,S3 |
| MI07 | 44 | F | 26.7 | 2 | N | Surgical | Ustekinumab, metformin, clindamycin, rifampicin | Crohn’s disease, hypertension | 2,S3 |
| mi08 | 28 | F | 21.4 |  |  | Surgical | Nil | Nil | 2,S3 |
| mi09 | 56 | F | 26.2 | 1 | Y | Surgical | Nil | Asthma, hypothyroidism | 2,S3 |
| mi10 | 19 | M | 23.8 | 1 | Y | Surgical | Lymecycline | Nil | 2,S3 |
| bk01 |  | F |  |  |  | Blood |  |  | 3 |
| BK06 | 45 | F | 36.7 |  | Y | Blood | Infliximab IV |  | 3 |
| BK11 | 42 | F | 31.9 |  | Y | Blood | Adalimumab | Nil | 3 |
| BK40 |  |  |  |  |  | Blood | Nil | Nil | 3 |
| BK42 | 39 | F | 46.5 |  | Y | Blood | Amjevita | Nil | 3 |
| HSM08 | 26 | F | 22.3 | 2 | N | Blood | Tetracycline | Hand dermatitis | 3 |
| HSM09 | 39 | F | 33.7 | 2 | Y | Blood | Nil | Depression, anxiety | 3 |
| HSM14 | 29 | F | 33 | 1 | Y | Blood | Nil | Nil | 3 |
| HSM28 | 46 | M | 26.3 | 2 | Y | Blood | Dapsone, eumovate | Nil | 3 |
| HSM31 | 39 | F | 33.2 | 2 | N | Blood | Nil | Nil | 3 |
| HSM33 | 47 | F | 38.3 | 2 | N | Blood | Duloxetine, Olmesartan, amlodipine | Depression, hypertension | 3 |
| BK04 | 41 | M | 27.6 |  | N | Blood | Metformin | Recurrent urinary tract infections | 3 |
| BK56 | 47 | F | 46.3 | 2 | Y | Blood | Metformin, OCP carpidopa/levodopa, PPI, antidepressant, ropinirole, antihistamine, rasagiline | Parkinson’s, polycystic ovarian syndrome, depression, anxiety, endometriosis | 3 |
| bk59 | 29 | F | 36.2 | 2 | Ex | Blood | Metformin, fluoxetine, amlodipine | Depression, anxiety, hypertension, pilonidal sinus | 3 |
| bk60 | 47 | F | 25.1 | 1 | Y | Blood | Metformin, lymecycline, lansoprazole | Heart block, pacemaker, heartburn | 3 |
| bk62 | 30 | F | 31.2 | 2 | Y | Blood | Metformin, spironolactone | Nil | 3 |
| bk63 | 52 | F | 44.8 | 2 | N | Blood | Metformin, lansoprazole | Type 2 diabetes, prev. brain tumour | 3 |
| hsm03 | 36 | F | 44.7 | 2 | Ex | Blood | Metformin, sertraline | Depression | 3 |
| hsm11 | 42 | F | 26.3 | 2 | Ex | Blood | Metformin, spironolactone | Nil | 3 |
| BK57 | 35 | F | 27.5 | 2 | Ex | Blood | Nil | Nil | 4 |
| BK58 | 30 | F | 46.5 | 2 | Y | Blood | Nil | Nil | 4 |
| BK61 | 28 | F | 29.7 | 2 | Y | Blood | Inhalers, sertraline, tramadol, OCP | Depression, anxiety, asthma, proximal kinesiogenic choreoathetosis | 4 |
| bk49 | 46 | F | 33.5 | 3 | Ex | Blood | Nil | Nil | 4 |
| BK50 | 31 | F | 35.7 | 2 | Y | Blood | Nil | Nil | 4 |
| BK53 | 32 | F | 32.2 | 2 | N | Blood | Ramipril, spironolactone | Nil | 4 |
| BK54 | 33 | F | 47.5 | 2 | Ex | Blood | Pantoprazole, venlafaxine, tetralysal, folic acid, vit D, OCP, telfast | Depression | 4 |
| BK55 | 30 | F | 50.7 | 1 | Y | Blood | Sertraline | Depression | 4 |
| MI23 | 37 | F | 29.8 | 3 | Y | Surgical | Rifampicin, clindamycin, lymecycline | Nil | 4 |
| MI24 | 69 | F | 19.5 |  | Y | Surgical | Infliximab | Crohn’s disease, osteoporosis | 4 |
| MI25 | 31 | M | 30 | 3 | Y | Surgical | Ponstan, auricalm | Nil | 4 |
| mI26 | 47 | M | 32.4 | 3 | Y | Surgical | Atecor, lymecycline | Hypertension | 4 |
| BK68 | 41 | M | 22.3 | 2 | Ex | Blood | Nil | Nil | 6 |
| BK69 | 30 | F | 30.2 | 2 | N | Blood | Lymecycline | Nil | 6 |
| BK70 | 20 | F | 26.6 | 2 | N | Blood | Nil | Nil | 6 |
| BK71 | 44 | F | 28.2 | 1 | Y | Blood | Nil | Nil | 6 |
| BK72 | 32 | F | 29.1 | 2 |  | Blood | Nil | Nil | 6 |
| bk91 | 48 | F | 31.9 | 2 | Y | Blood | Nil | Nil | 6 |
| bk93 | 32 | F | 59.7 | 2 | N | Blood | Cannabis, tetralysal, oteupic | Obesity, depression, asthma, anxiety, autism, hypertension, fibromyalgia, Barrett’s oesophagus | 6 |
| bk94 | 19 | M | 36.9 | 2 | N | Blood | Nil | Acne, obesity | 6 |
| bk95 | 38 | F | 30.8 | 3 | Y | Blood | Nil | Asthma, obesity, osteoarthritis | 6 |
| bk96 | 63 | M | 31.2 | 2 | Y | Blood | Metformin | Type 1 diabetes | 6 |
| bk97 | 24 | M | 32.9 | 2 | N | Blood | Spironolactone | Acne, recurrent orbital cellulitis | 6 |
| bk98 | 40 | F | 31.9 | 2 | Y | Blood | Nil | Nil | 6 |
| mi11 | 33 | F | 39.2 | 3 | Y | Surgical |  | PCOS, asthma, inflammatory bowel disease | S2 |
| mi12 | 29 | F | 29 | 2 | Y | Surgical |  | Psoriasis | S2 |
| mi13 | 31 | M | 29.9 | 3 | N | Surgical | Nil | Nil | S2 |
| mi17 | 38 | M |  |  | Ex | Surgical | Tetracycline | Acne, asthma, gastro-oesophageal reflux disease | S2 |
| mi18 | 39 | F | 37 | 3 | Ex | Surgical | Guselkumab | Type 2 diabetes | S2 |
| MI14 | 35 | F | 41.7 | 2 | Y | Surgical | Adalimumab | Type 2 diabetes, asthma, hypothyroidism | S2 |
| MI15 | 42 | F | 31.5 | 2 | Y | Surgical |  | Depression, anxiety | S2 |
| BK52 | 20 | F | 33.2 | 2 | N | Punch | Venlafaxine, spironolactone, tetralysal | Depression, anxiety, hole in heart | S2 |

**Table S3.** Psoriasis patients

| ID | Age (Y) | Sex | BMI | PASI | Smo-  ker | Sample type | Medications | Comorbidities | Fig |
| --- | --- | --- | --- | --- | --- | --- | --- | --- | --- |
| PSO2 | 29 | M | 20.5 | 6.4 | N | Punch | Nil | PSA | S1 |
| PSO3 | 66 | M | 32.7 | 8.3 | Ex | Punch | Statin, antihypertensives | Type 2 diabetes, hypercholesterolemia, hypertension | S1 |
| PSO5 | 49 | M | 29.3 | 6.1 | N | Punch | Nil | Nil | S1 |
| PSO6 | 49 | F | 32 | 7.7 | Ex | Punch | Lamotrigine, vortioxetine, esomeprazole, zolpidem mirtazapine,promethazine | Bipolar affective disorder, inflammatory bowel syndrome, fatty liver | S1 |
| PSO7 | 21 | M | 20.6 | 5.2 | N | Punch | Nil | Hypertrophic cardiomyopathy | S1 |
| PSO9 | 38 | M | 33.2 | 14.8 | Ex | Punch | Nil | Nil | S1 |
| PSO10 | 29 | F | 31.2 | 6 | N | Punch | Nil | Nil | S1 |

**Table S4.** Demographics for patients and healthy controls

| Healthy Controls | | | | | |
| --- | --- | --- | --- | --- | --- |
| Figure | N number | M:F ratio | Age range | Mean age | Mean BMI |
| 3 | 7 | 2:3 | 24-44 | 31.6 | 23.4 |
| 6 A | 6 | 1:1 | 25-34 | 30.5 | 24.3 |
| 6 B | 5 | 2:3 | 23-46 | 34 | 25.3 |
| 6 C-D | 6 | 7 |  |  |  |
| S2 A-C | 8 | 0:10 | 42-69 | 53.5 |  |
| S2 D | 3 | 0:10 |  |  |  |
| Hidradenitis suppurativa patients | | | | | |
| Figure | N number | M:F ratio | Age range | Mean age | Mean BMI |
| 1, 5 A | 10 | 3:7 | 23-52 | 34.9 | 37.2 |
| 2, 5 B-D, S3 | 10 | 1:9 | 19-58 | 39 | 37.17 |
| 3 A-D | 11 | 1:9 | 26-47 | 39.1 | 33.6 |
| 3 E | 10 | 0:10 | 28-47 | 35.1 | 37.5 |
| 4 | 4 | 1:1 | 31-69 | 46 | 27.9 |
| 6 A | 6 | 1:2 | 19-48 | 33.5 | 37.3 |
| 6 B | 5 | 1:4 | 20-44 | 33.4 | 27.3 |
| S2 A-C | 6 | 3:7 | 22-39 | 32 | 37.2 |
| S2 D | 3 | 0:10 | 20-42 | 32.3 | 38.8 |
| Metformin treated Hidradenitis suppurativa patients | | | | | |
| Figure | N number | M:F ratio | Age range | Mean age | Mean BMI |
| 1, 5 A | 10 | 0:10 | 22-60 | 34.8 | 36.5 |
| 3 | 10 | 1:9 | 22-52 | 37.9 | 34.9 |
| Psoriasis patients | | | | | |
| Figure | N number | M:F ratio | Age range | Mean age | Mean BMI |
| S1 | 7 | 7:3 | 21-66 | 40.1 | 28.5 |

**Table S5.** Primer sequences for RT-qPCR using SYBR Green

| Target | Name | Direction | Sequence |
| --- | --- | --- | --- |
| IL-17A | Interleukin 17 A | For | 5’-AAG ACC TCA TTG GTG TCA CTG C-3’ |
|  |  | Rev | 5’-ATT GTG ATT CCT GCC TTC ACT ATG-3’ |
| IFN-γ | Interferon gamma | For | 5’-TTG AAG AAT TGG AAA GAG GAG AGT G-3’ |
|  |  | Rev | 5’-AAA GGA GAC AAT TTG GCT CTG CAT T-3’ |
| TNF-α | Tumour necrosis factor A | For | 5’-ACC TCT CTC TAA TCA GCC CTC-3’ |
|  |  | Rev | 5’-GGT TCG AGA AGA TGA TCT GAC TG-3’ |
| IL-6 | Interleukin 6 | For | 5’-CCC TGA GAA AGG AGA CAT CTA AC -3’ |
|  |  | Rev | 5’-CCC TGA GAA AGG AGA CAT CTA AC -3’ |
| GLUT1 | Glucose transporter 1 | For | 5’-CTT CGA GTA TGT GGA GCA ACT GT-3’ |
|  |  | Rev | 5’-GCA CAG TGA AGA TGA TGA AGA CG-3’ |
| HK2 | Hexokinase 2 | For | 5’-TTC TTG TCT CAG ATT GAG AGT GAC-3’ |
|  |  | Rev | 5’-TTG CAG GAT GGC TCG GAC TTG-3’ |
| PFKFB3 | Phosphofructokinase B3 | For | 5’-GGC AGG AGA ATG TGC TGG TCA T-3’ |
|  |  | Rev | 5’-CAT AAG CGA CAG GCG TCA GTT TC-3’ |
| RPLP0 | Ribosomal protein lateral stalk subunit P0 | For | 5’-GCG TCC TCG TGG AAG TGA CAT CG-3’ |
|  |  | Rev | 5’-TCA GGG ATT GCC ACG CAG GG-3’ |
